# Implementation of Complex Biological Logic Circuits Using Spatially Distributed Multicellular Consortia

**DOI:** 10.1371/journal.pcbi.1004685

**Published:** 2016-02-01

**Authors:** Javier Macia, Romilde Manzoni, Núria Conde, Arturo Urrios, Eulàlia de Nadal, Ricard Solé, Francesc Posas

**Affiliations:** 1 ICREA-Complex Systems Laboratory, Universitat Pompeu Fabra (UPF), Barcelona, Spain; 2 Cell Signaling Research Group, Departament de Ciències Experimentals i de la Salut, Universitat Pompeu Fabra (UPF), Barcelona, Spain; 3 Santa Fe Institute, Santa Fe, New Mexico, United States of America; Columbia University, UNITED STATES

## Abstract

Engineered synthetic biological devices have been designed to perform a variety of functions from sensing molecules and bioremediation to energy production and biomedicine. Notwithstanding, a major limitation of *in vivo* circuit implementation is the constraint associated to the use of standard methodologies for circuit design. Thus, future success of these devices depends on obtaining circuits with scalable complexity and reusable parts. Here we show how to build complex computational devices using multicellular consortia and space as key computational elements. This spatial modular design grants scalability since its general architecture is independent of the circuit’s complexity, minimizes wiring requirements and allows component reusability with minimal genetic engineering. The potential use of this approach is demonstrated by implementation of complex logical functions with up to six inputs, thus demonstrating the scalability and flexibility of this method. The potential implications of our results are outlined.

## Author Summary

Synthetic biological circuits have been built for different purposes. Nevertheless, the way these devices have been designed so far present several limitations: complex genetic engineering is required to implement complex circuits, and once the parts are built, they are not reusable. We proposed to distribute the computation in several cellular consortia that are physically separated, thus ensuring implementation of circuits independently of their complexity and using reusable components with minimal genetic engineering. This approach allows an easy implementation of multicellular computing devices for secretable inputs or biosensing purposes.

## Introduction

Synthetic biological devices have been built to perform a variety of functions [1–3]. Currently, the creation of complex logic circuits capable of integrating a high number of different inputs and of performing non-trivial decision making processes is one of the major challenges of synthetic biology [4–8]. Examples of synthetic gene circuits used to perform digital computation are switches [9,10], logic gates [11,12], oscillators [13], band-pass filters [14], classifiers [15] and memory devices [16]. However, despite the enormous efforts devoted to developing such devices, the results obtained are far from the level of complexity needed for applications [17,18]. Limitations in the design of some of these devices and the lack of reusability of the genetic modules strongly constrains the degree of scalability and complexity necessary for industrial, environmental or biomedical applications [19].

In general, the implementation of biological devices that are capable of performing complex logical computations in response to a growing number of input signals involves complex genetic engineering with limited reusability. Usually, the circuits are obtained (or designed) by connecting basic logic gates following standard combinatorial logic, inspired by the circuit analogies applied to understanding genetic networks [20–24]. Dedicated efforts have been oriented towards the exploration of such combinatorial scheme within synthetic biology [25,26]. In accordance with this standard architecture, the functional complexity of a circuit will scale up with both the number of different logic gates and the number of wires that connect them (i. e. circuit connectivity). Both elements limit the scalability and complexity of these devices [19]. One of the most restrictive constraints is the so-called wiring problem. While wiring is not a major problem in standard electronics, in biological systems is a key limiting factor. This limitation arises from the fact that each connection (wire) requires a different biochemical entity and that crosstalk needs to be prevented [27].

In spite of the efforts aimed at standardization of genetic components in synthetic biology, serious limitations still exist [28]. This limits both scalability and the potential reuse of genetic components. Along with the wiring problem, novel strategies towards synthetic biological computation seem required to overcome these problems. In this context, the implementation of circuits using multicellular consortia instead of single cells allows for a reduction in the genetic engineering required in a particular cell [29,30] and the reusability of the components. In this scenario, each cell carries a particular engineered design that, when combined with other cells of the consortia, performs the final computation (hereafter *distributed* computation) [27]. Furthermore, when this approach is combined with distributed output production [31] or spatial segregation [32] it allows the attainment of logic circuits with a significant reduction in the number of wires and genetic manipulations required. Noteworthy, both in nature and engineering, space is used as an added dimension of information processing, such as in intracellular network computation [33], amorphous computing [34], cell-cell interaction [35], pattern formation [36–38], or in ant colonies [39,40]. Nevertheless, spatial segregation has never been fully exploited as a key computational parameter in the building of synthetic biological devices [17–19].

Here we present a novel methodology that allows designing biological devices based on the combination of three elements: multicellular consortia, distributed output production and spatial segregation. A major reason to adopt this approximation is the division of labor already present in tissues and organs, where different cell types perform different functions while communicating through signaling molecules. Such segregation of functions, combined with integration of signals is a universal design principle of multicellular systems. Our approach uses engineered cells (our *cell types*) implementing one-input one-output logic gates organized in several consortia, connecting cells of each consortium with a single wire, and allowing each consortium to produce the final output independently of the others. This systematic and simplified distribution of the computation, together with spatial isolation in modules of the different multicellular consortia, permits the building of complex logic circuits. Modules and cells can be reorganised to obtain different computations. Notably, only one wiring molecule and minimal genetic engineering is used. An important property of this architecture is that it does not depend on the complexity of the circuit to be implemented; thus, scalability is ensured because the required number of cell types and modules are bounded. As a proof of principle, we have built several logic circuits in eukaryotic cells with increasing complexity that respond to up to 6 inputs (such as a 4-to-1 multiplexer). Furthermore, we focused on two particular, but very relevant, types of biological devices with diverse outputs. The first type of devices produce an output with a determined function that is secreted into the medium (e.g. hormone, secretable enzyme). Such devices could be used in bioreactors for the production of enzyme, metabolites, or recombinant proteins, as well as in biomedical industry for the production of pharmaceutical products like hormones or drugs. The second type, called transducers (e.g. biosensors) transform a combination of different external inputs into a single signal that can be easily quantified with reader devices. Those are devices that could be used for example in diagnostic kits, microbiological assays or biodetectors. In both scenarios, this novel architecture allows the construction of modular biocomputers in a flexible, robust and scalable manner.

## Results

### Logic multicellular circuits with distributed computation show reduced wiring requirements

With the aim of reducing wiring requirements and minimizing *in vivo* genetic manipulations, we designed a new logic architecture for use in biological circuits. The basis of this architecture is the combination of multiple consortia with distributed computation [31] with the use of spatial confinement [32]. In general, the behavior of a given logic circuit responding to N inputs can be defined by a logic Boolean function described by the so-called truth table. This table defines all possible combinations of inputs and the associated outputs. According to Boolean algebra, the so-called canonical form of the Boolean function can also describe the truth table of a given logic circuit. Independently on the particular circuit analyzed, this canonical form follows the general expression:

$$f=\overset{M}{\underset{i=1}{\sum }}\left[\overset{N}{\underset{j=1}{\prod }}\phi_{ij}(x_{j})\right]$$

Here Σ represents the OR operator and Π the AND operator. The function *ϕ*_*ij*_ is either a logic representation of the presence of a molecular input *x*_*j*_ (Identity function) or of its absence (NOT function). Finally, *M* is the maximum number of terms present in the Boolean function, which depends on the complexity of the function, but the condition *M* ≤ 2^N-1^ is always satisfied [41]. In general, the expression of a Boolean function *f* can be reduced by the systematic application of standard rules of simplification, such as Karnaugh maps [42] or the Quine-McCluskey algorithm [43]. However, in the biological context easier implementations can be achieved modifying the canonical expression of the Boolean function to obtain an expression involving only OR logic (the simpler logic in a cellular implementation). To reach this goal we propose an alternative formulation of the Boolean function based on the Inverted Logic Formulation (ILF). This formulation minimizes biological constraints ensuring scalability (see S1 Text for a detailed mathematical description).

The canonical form of a general Boolean function can be rewritten applying a double negation, i.e.

$$f=\bar{\bar{f}}=\bar{\bar{\overset{M}{\underset{i=1}{\sum }}\left[\overset{N}{\underset{j=1}{\prod }}\phi_{ij}(x_{j})\right]}}$$

Applying the Morgan’s Laws [44],

$$\left\{ \begin{array}{l} \bar{a\overset{}{}\;OR\;\overset{}{}b}=\bar{a}\overset{}{}\;AND\;\overset{}{}\bar{b}\\ \bar{a\overset{}{}\;AND\;\overset{}{}b}=\bar{a}\overset{}{}\;OR\;\overset{}{}\bar{b} \end{array}\right.$$

the Boolean function can be expressed as $f=\bar{\bar{f}}=\sum_{i=1}^{M}\left[\bar{\sum_{j=1}^{N}\theta_{ij}(x_{j})}\right]$ where $\theta_{ij}(x_{j})=\bar{\phi_{ij}(x_{j})}$ (see S1 Text for a detailed mathematical description). Hence, the Boolean function results in the OR combination of several computational modules *ψ*_*i*_, i.e.: $f=\sum_{i=1}^{M}\psi_{i}$

Each module *ψ*_*i*_ is the inversion of OR combinations (symbol Σ) of inverted terms *θ*_*ij*_, i.e. $\psi_{i}=\bar{\sum_{i=1}^{N}\theta_{ij}(x_{j})}$ where *θ*_*ij*_*(x*_*j*_*)* can be chosen among NOT or Identity functions, i.e.

$$\theta_{ij}(x_{j})=\{\begin{array}{c} \bar{x_{j}}\\ or\\ x_{j} \end{array}$$

depending on the specific function to be implemented by the circuit.

**Fig 1:**
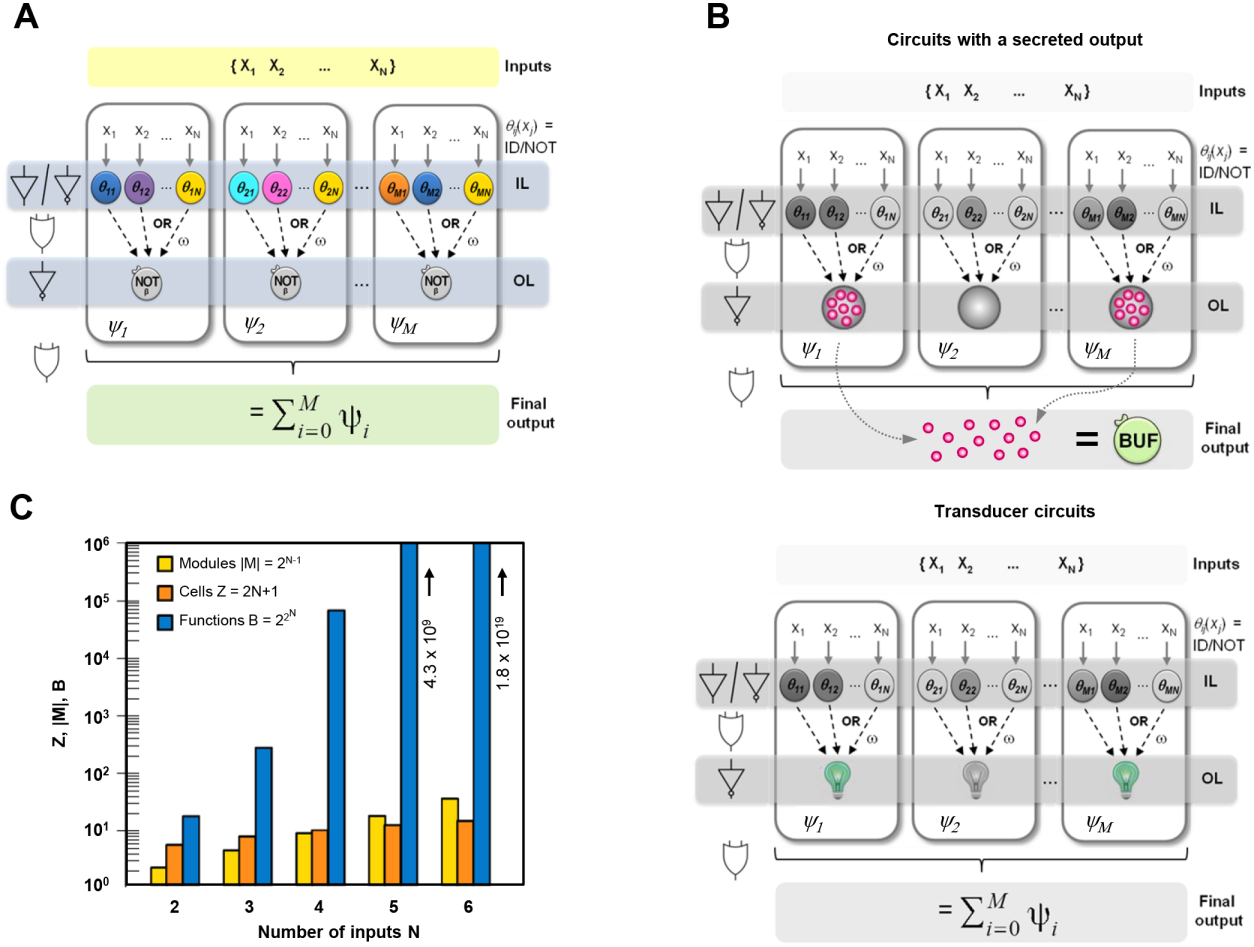
Schematic diagram of modular distributed computation and the scalability of this architecture in logic circuits. (A) Schematic diagram of spatially distributed computation. A spatially organized set of chambers defining a set of modules provides a source of modularity by separating different subsets of cells (consortia) into different groups. Each module contains one or more cells from the Input Layer (IL) library (that respond according to an ID or NOT logic), all of which sense only one signal from a given repertoire of N inputs (X) and respond by secreting a communication molecule ω. All modules also include Output Layer (OL) cells that produce the output β implementing a NOT logic, in response to molecule ω. The concentration of β can be quantified directly from the module if the OL is a reporter cell and then the output of the whole circuit (Final output) is the OR combination of each consortium output. Alternatively, the final output can be quantified by using an optional Buffer Layer (BL) cell (BUF) that integrates the final outcome from the different modules if β is a secretable molecule. (B) Schematic diagram explaining the differences between a circuit with a secreted output (upper panel) and transducer circuits (bottom panel). The buffer cell (BUF) collects the output of the different chambers and produces the final computation (top). (C) Illustration of the combinatorial potential of spatially restricted distributed computation architecture. The plot shows the required number of cells, the number of modules and the number of potential N-input logic functions against the number of inputs. Spatial modules (M) and cells number (Z) scale slowly with the number of inputs (N), whereas the repertoire of logic functions shows super exponential increases even for small N.

This formalism can be easily translated into a biological implementation. Fig 1 shows a schematic representation of the proposed architecture. In the general basic design, a particular logic circuit is composed of M different multicellular consortia (Fig 1A) located in physically isolated chambers {*ψ*_*1*_, *ψ*_*2*_,*… ψ*_*M*_}. Here, each module *ψ*_*i*_ that conform the Boolean function can be biologically implemented by a different multicellular consortia located in a physically isolated chamber. Each consortium contains two different layers of cells, namely the Input Layer (IL) and the Output Layer (OL). The Input Layer is composed of several cell types that sense single external inputs {x_*1*_, x_*2*_,*…x*_*N*_} and secretes a wiring molecule ω according to a particular internal logic, Identity (ID) or NOT (NOT) logic implementing the *θ*_*ij*_*(x*_*i*_*)* functions. When the wiring molecules ω secreted by each cell are mixed in the medium, the OR function (Σ) is implicitly implemented.

The OL consists in a single cell type that responds to the wiring molecule (ω) producing the output β according to a NOT logic, i.e. the output molecule (β) is produced only in the absence of the wiring molecule (ω). Of note, only two types of elementary logic responses are implemented in engineered cells (ID or NOT), yet the logic circuitry is much more sophisticated thanks to the spatial segregation of the consortia. In each consortium, the IL cells produce the same wiring molecule in a shared environment thus implementing an implicit OR logic gate. Combining this OR gate with the NOT gate of the OL cells results in a multi-input NOR gate. Remarkably, only one wire (ω) is needed. The output of the whole circuit is the OR combination of each consortium output. This OR combination can be easily implemented by connecting the chambers that contain each consortium and mixing the output (β) produced using an integrated device. Hence this architecture is optimal for systems where the output is a secreted molecule (e.g. hormone or enzyme). Because the circuit is based on distributed computation, in the presence of a given combination of inputs, the output (β) can be produced in one or more consortia at the same time. Therefore the final output concentration can be different depending on the number of consortia producing it. Despite being a digital approach, in which only the presence (logic state 1) or the absence (logic state 0) of the output molecule is relevant, in a real applied system the total amount of output production could be meaningful. Hence, the total amount of output production should not be dependent on the specific combination of inputs that induces their production. This problem can be solved introducing a buffer cell (BUF cell) that senses β secreted by the different consortia and produces the final output of the circuit according to identity logic. This buffer cell has to be designed that responds at maximum when senses the presence of the output signal (β) from a single consortium. Hence, higher levels of β will not be translated into differentiated output levels (Fig 1B, upper panel). Alternatively, in devices where β is a simple readout of the computation like “transducer circuit” (e.g. biosensors), the final OR could be assessed by quantification of a reporter (e.g. fluorescence) directly expressed in the OL cells using a reader device (e.g. FACS or microscopy). In these cases, a positive signal in any consortia can account as the final positive output of the computation bypassing the need for an integrated device and a buffer cell (Fig 1B, bottom panel).

The main feature of this architecture is its general design, i.e. for a given number (N) of inputs any arbitrary circuit can be built by using the same architecture, independently of its complexity. A simple calculation reveals that, while the upper bound size of the cell library necessary to implement circuits integrating N different inputs scales linearly as Z = 2N+1 (ID and NOT for each input plus the OL cell) and the maximum number of spatial modules (*¬*_*i = 1*,*…M*_) increases according to M = 2^N-1^, the number of implementable different logic functions *B* grows super-exponentially as $\text{B }=\;2^{2}{}^{{}^{N}}$ (Fig 1C). For instance, to implement all the 5-input functions (B = 4.294.967.296), only 11 different cell types and, in the most complex scenario, 16 independent modules (M = 16) are needed. Of note, we are here referring to a single output (β) circuit. In multiple-output circuits the upper bound on the number of OL cells is equal to the number of circuit’s output. When using the optional Buffer layer (BL) only one cell type and one additional module are required. Notably, the simple combination of ID and NOT logic cells, when spatially segregated, defines a *functional complete set* that guarantees that any logic circuit can be built by combining these elements (a formal demonstration of this design and a detailed description of a systematic methodology for logic circuit implementation are included in S1 Fig and in S1 Text. Therefore, any arbitrary logic function can be encoded in a number M of different consortia and in the particular combinations of IL cells in each of these consortia (Z) (Fig 1C). Increasing functional complexity of the logic circuits is translated into an increase in the number of consortia and the corresponding chambers, but not in the number of cell types or wires. In order to demonstrate that our architecture allows scalability together with the minimization of the genetic engineering requirements, we built several logic circuits in eukaryotic cells that respond to up to 6 inputs.

### A library of cells with minimal genetic manipulation serves to create *in vivo* multicellular consortia that specifically respond to different inputs

**Fig 2:**
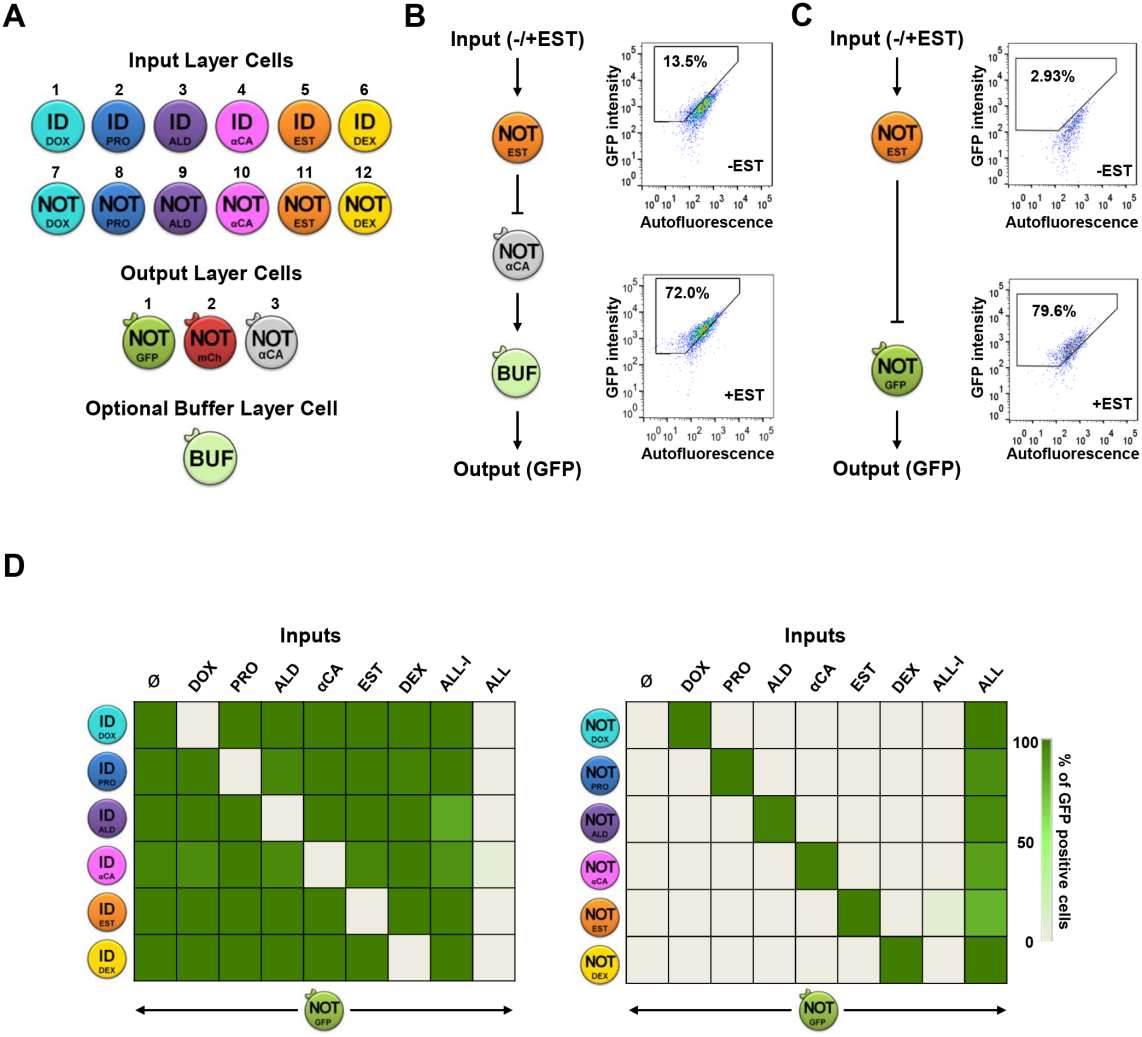
A library of engineered cells for implementation of complex biological circuits. (A) Schematic representation of the Input (IL), Output (OL) and Buffer (BUF) Layer cells library. Each color indicates cells that respond to a different input with Identity (ID) or NOT logic (see Materials and Methods and S1 Text for a complete cell library description and characterization). (B) Quantification of single cell computational output with the optional BUF cells. IL cells (#11) stimulated with 17-β-estradiol (EST) was mixed with OL_3_ cells in the absence (-EST) or presence (+EST) of the input (left). After 4 h of computation the supernatant of the mix was added to the BL cells, incubated for 4 h and the percentage of GFP-positive cells was analyzed using FACS (right). (C) Quantification of single cell computational output without the optional Buffer Layer cells. IL cells (#11) were stimulated with 17-β-estradiol (EST) and were mixed with OL_1_ cells in the absence (-EST) or presence (+EST) of the input (left). After 4 h of computation the percentage of GFP-positive cells was analyzed using FACS (right). (D) Crosstalk analysis of IL cells (ID, left; NOT, right). IL cells were mixed with OL_1_ cells (GFP) in the presence of all 6 inputs individually, all 6 inputs together (ALL), or all inputs except for the specific one (ALL-I). Results are expressed as a percentage of GFP-positive cells.

In order to implement modular biocomputing *in vivo*, we created a minimal library of engineered yeast cells required for the ILs and OLs of the circuits (Fig 2A and S2–S4 Figs). The IL library consists of six pairs of cells that respond to extracellular stimuli, namely: doxycycline (DOX), progesterone (PRO), aldosterone (ALD), mating α-factor from *C*. *albicans* (αCa), 17-β-estradiol (EST) and dexamethasone (DEX). The detection of those hormones is done by expressing the specific receptor in each cell type (see full description in S1 Text). Each pair of cells consists of two different types of cells that respond to the same stimulus but with a different logic, either ID or NOT logic, and that secrete a wiring molecule, the *S*. *cerevisiae* α-factor pheromone (αSc) accordingly. ID cells secrete the wiring molecule upon stimulation by expressing the *MF(*α*)1* gene from a specific promoter that responds to a defined stimulus. The corresponding pair-wise cell with the NOT logic express the LacI repressor from the same stimulus-specific promoter. NOT cells express the *MF(*α*)1* gene under a modified *TEF1* promoter that contains LacI binding sites (P_*TEF1i*_) and thus, inhibits αSc expression in the presence of stimuli (Fig 2A and S2A Fig).

Cells in the OL respond to the αSc that is secreted by IL cells, and subsequently express, or do not express a protein (β) (the output of the module), according to NOT logic. In the general architecture, β can be a secreted molecule that performs a determined function (e.g. an enzyme or a hormone) or a fluorescent protein (i.e. GFP or mCherry). Briefly, OL able to secrete molecules (OL_3_) consist in a cell that constitutively expresses the *C*. *albicans* α–factor (αCa) gene, *CaMF(a)1*, under the *TEF1i* promoter. The αCa is secreted in the culture media mimicking hormones in biomedical application or protein production in bioreactors. The LacI repressor is transcribed from the *FUS1* promoter. Therefore, in the presence of *S*. *cerevisiae* α-factor (i.e. the wiring molecule) the LacI repressor is produced and represses the expression of *C*. *albicans* α-factor (Fig 2A and S2C Fig). Alternatively, fluorescent OL cells (OL_1_ and OL_2_) constitutively express a modified version of a fluorescent protein (yEGFP or mCherry) fused to a degradation tag (ssrA) under the *TEF1i* promoter [45]. The presence of the wiring molecule induces LacI expression, which leads to down-regulation of fluorescent protein expression. Pheromone (αSc) also stimulates degradation of the fluorescent protein by induction of the ClpXP protease complex that recognizes and degrades ssrA-tagged proteins (Fig 2A and S2B Fig).

Given that the output of the circuit is distributed in different consortia the concentration of the secreted molecule (e.g. αCa) can differ according to the number of consortia simultaneously producing it. In case that the level of the secreted molecule needs to be constrained within given thresholds, we engineered a Buffer Layer cell (BUF), which is designed to produce GFP in the presence of αCa according to Identity logic. This cell contains *GFP* integrated into the *FUS1* gene locus under its promoter (*FUS1*::*GFP-KanMX)*. BL cells also express the *C*. *albicans* pheromone receptor (*CaSTE2*) so that they can sense the secreted pheromone (Fig 2A and S2D Fig). The buffer cell has been designed to give the maximum response when it detects αCa from a single consortium in a sharp step-like function (S7B Fig). Hence, higher levels of αCa will not be translated into different output levels. The genotype and the graphical notation of the logic function performed by each cell (Input, Output and Buffer Layers) of the library are depicted in Fig 2 and S3 and S4 Figs and in S1 Text.

Once the library of cells was built, we coupled one cell from the IL with one of the OL in the presence or in the absence of the input in order to demonstrate that the wire connection works properly. Two possible scenarios are described: computation of the IL cells in the presence (Fig 2B) or the absence (Fig 2C) of the optional BL cells. Similar output results were obtained using both strategies by measuring the fluorescence of single cells using flow cytometry. The autofluorescence and the percentage of cells able to produce a positive output signal that were fluorescent positive cells were calculated (S5 Fig). These results showed a clear separation between 0 and 1 logic states in the response of the cells.

We then extended the analysis to all of the cell types by measuring their transfer function (i.e. the relationship between different input concentrations and the corresponding output production) (S6 and S7 Figs). Briefly, the transfer function of OL cells was characterized by incubation with increasing concentrations of the input synthetic αSc and measurement of the output fluorescence by FACS (OL_1_ and OL_2_), or the output fluorescence resulting from secretion of the αCa (OL_3_) after incubation with BUF cells. Similarly, the transfer function of the BL cells was characterized by incubation with increasing concentration of synthetic αCa. The transfer function of IL cells was assessed by measurement of output fluorescence upon exposure to increasing levels of each stimulus in the presence of the OL_1_ cells. These experimental results indicate that all of the cells exhibit a proper behavior that allows definition of a clear separation between 0 and 1 logic states. Applying the same methodology used in electronics, we defined a threshold. Cells producing an output below the threshold are in the 0 logic state, whereas if the output is above this threshold is considered in the 1 logic state. This threshold is the same for all the cells and circuits analyzed.

Based on these results, we established the concentration of inputs used in the circuits (see below) so that they were clearly above the threshold in order to guarantee a correct response of the cells. Also, based on these transfer functions results, we determined a specific range of time for the response to input signals used in the circuits to ensure a robust response. When working with cellular consortia, it is critical for the system to work robustly that cells within a consortium display similar growth rates. Thus, we assessed the growth rate of each cell type and found no major differences within the entire cell library (S8 and S9 Figs) suggesting that the different consortia should not display unbalanced cell growth of any of the components.

A potential thread to implement complex biological circuits is crosstalk between cells. We therefore assessed crosstalk between the IL cells in response to different single inputs or to all of the inputs combined. Each IL cell type was mixed with OL_1_ cells and then treated separately with every input. The percentage of GFP positive cells was measured using FACS. Each cell type responds only to its own stimulus and secretes the pheromone only in the presence (ID) or in the absence (NOT) of the specific input. Finally, we incubated every cell type with all of the inputs to which it should not respond (ALL-I). Even in this scenario, no significant crosstalk was observed (Fig 2D). Therefore the crosstalk between the IL cells upon different inputs was not significant.

We then implemented a number of 2-input logic gates to test the combination of several cell types from the library. Here, just to test the cells we measured the output of the logic circuits as the GFP production of the OL_1_ cell (S10 Fig) and found that the cells computed correctly when an AND, NOR or N-IMPLIES gates were assessed.

### Combinatorial modular organization of a minimal cell library permits the implementation of complex logic circuits

**Fig 3:**
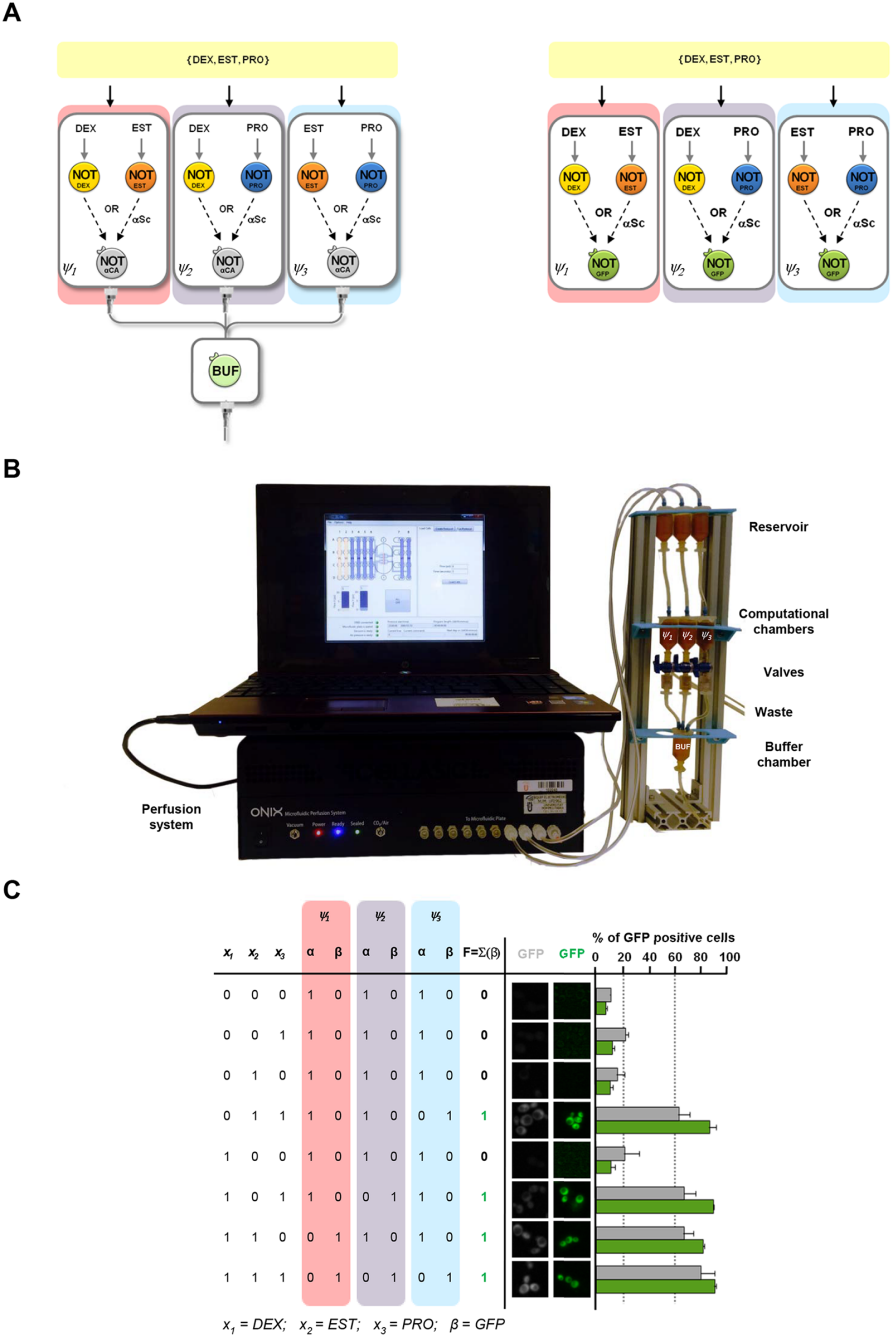
Design and *in vivo* implementation of a 3-input majority rule. (A) Schematic representation and spatial organization of the cells in the majority rule using OL_3_ and the optional Buffer Layer cells (left), or OL_1_ (right). (B) Image of the open-flow computation device for one combination of inputs. The device contains three chambers that are connected to a final chamber that contains BL cells. The flow in the circuit is managed by an air pump. (C) Truth table (left), microscope images (middle) and percentage of GFP-positive cells (right). Cells were mixed proportionally in the corresponding chamber as described in Fig 3A. Different combinations of inputs (dexamethasone (DEX), 17-β-estradiol (EST) and progesterone (PRO)) were added at the same time to the three chambers. Grey refers to the implementation of all the combination of the majority rule circuits using the open-flow computation device. After computing, for each combination of inputs, valves were open and output liquid from the three chambers was collected into a BL cell chamber containing Buffer cells and let them compute for 4 h at 30°C. The percentage of GFP positive BL cells was analyzed using FACS and cells were also collected for microscopic analysis. Data represent the mean and standard error of three independent experiments. Green refers to the circuit implemented as a biosensor using OL_1_. Here, after computing, the percentage of GFP positive cells in each chamber was analyzed using FACS and cells were also collected for microscopic analysis. Data were analyzed and processed as described in Material and Methods and in S11 Fig and represent the mean and standard error of the chamber with highest % of positive cells from three independent experiments.

As an example of how this modular architecture works we built the majority rule device (Fig 3), by testing it in circuits with a secretable output. This three inputs circuit is a decision-making system used in electronics as a security device against failure in redundant systems. The formal representation of the circuits is shown in S14A and S14B Fig. Using our library of cells, we implemented it as a device that detects when at least two molecules out of three are present. Determine the unbalance between molecules concentrations could be of interests in biomedical applications. The equivalent, single-cell type design of a majority rule would be very difficult to build *in vivo* [46]. To define the best cell combination from the library within the different modules, the design of the logic circuit is first done *in silico* which ensure the use of the correct combination of cells (see S1 Text for a detail description of a systematic methodology for logic circuit implementation). Following the basic architecture described above, implementation required just three different multicellular consortia, *ψ*_1_, *ψ*_*2*_ and *ψ*_*3*_, formed combining three IL cell types with the OL_3_ cell. Production of the secretable molecule from the independent modules was sensed by the BL cell (Fig 3A, left).

A key element in the proposed architecture is the spatial segregation of the different modules. Here, the final OR computation is done physically connecting the modules and collecting the output (i.e. a secreted molecule). To this purpose we built an open-flow computing device (Fig 3B) with physically isolated chambers and able to collect and integrate the outputs (here αCa) from the different consortia of the circuit. The different consortia were assembled in three independent *computational* chambers (*ψ*_1_, *ψ*_*2*_ and *ψ*_*3*_) and exposed to the same combination of the three inputs (*x*_*1*_ = DEX, *x*_*2*_ = EST, *x*_*3*_ = PRO). The buffer cells were incubated in the *Buffer* chamber (BUF). After a transitory computational time, the device is programmed to gather the fluxes of αCa produced by the independent consortia in the *Buffer* chamber, thereby performing the final OR computation. IL cells were prevented to enter into the OR chamber by positioning a filter before the Buffer chamber. The final output of the circuit, stored in the *Buffer* chamber, was quantified as % of GFP positive BL cells using both microscopy and flow cytometry (Fig 3C, grey bars). All the eight possible combinations of inputs were tested and the final outcome of the computation was as expected for a majority rule circuit: only when at least two of the inputs were present there was a positive output.

The open-flow computing device is an example of an integrated system able to implement *in vivo* circuits with a secreted output. The second type of devices that can be implemented with the this architecture is the transducers circuits. These circuits are devoted to translate a complex combination of multiple external inputs into a single output signal. The architecture of transducer circuits is simpler because they do not require the final output integration. These circuits can be built as an array of separated modules (chambers) that produce the same fluorescent protein as an output which, in turns, is measured by an external readout system. More specifically, we assessed the output by direct quantification of the OL_1_ or OL_2_ fluorescence using microscopy and flow cytometry as reader devices. The final output of the circuit was considered positive whenever any consortia gave a positive fluorescent signal above the 1 logic state threshold, bypassing the need of a full system integration. As a first example of a transducer, we measured the output of the same majority rule circuit using OL_1_ instead of OL_3_ (Fig 3A, right) mimicking a biosensing circuit. Fig 3C, green bars, shows that the circuit responded similar to the open-flow computing device even using a different type of OL cell. Thus, a combination of cell types with the proper design resulted in a device that was capable of implementing a majority rule circuit *in vivo*. The final result of the computation of the circuit is given as % of GFP cells with fluorescence below (0 state) or above (1 state) the logic threshold previously defined. Alternatively, the output could be measured in terms of total GFP fluorescence (in arbitrary units). We demonstrate that for our library of cells, both metrics are qualitatively equivalent (S12 Fig and S1 Text). A detailed description of the measurement procedures and outcome circuit production is included in the Material and Methods section and S11 Fig. To show the flexibility and robustness of the cells library, we built the same circuit using IL_7_ cells, which respond to DOX, instead of IL_12_ cells, which respond to DEX. S13 Fig shows that the logic circuit responded similarly and reliably even swapping cells from the library to respond to different inputs with the same logic, indicating the robustness of the circuit response to cell variation.

**Fig 4:**
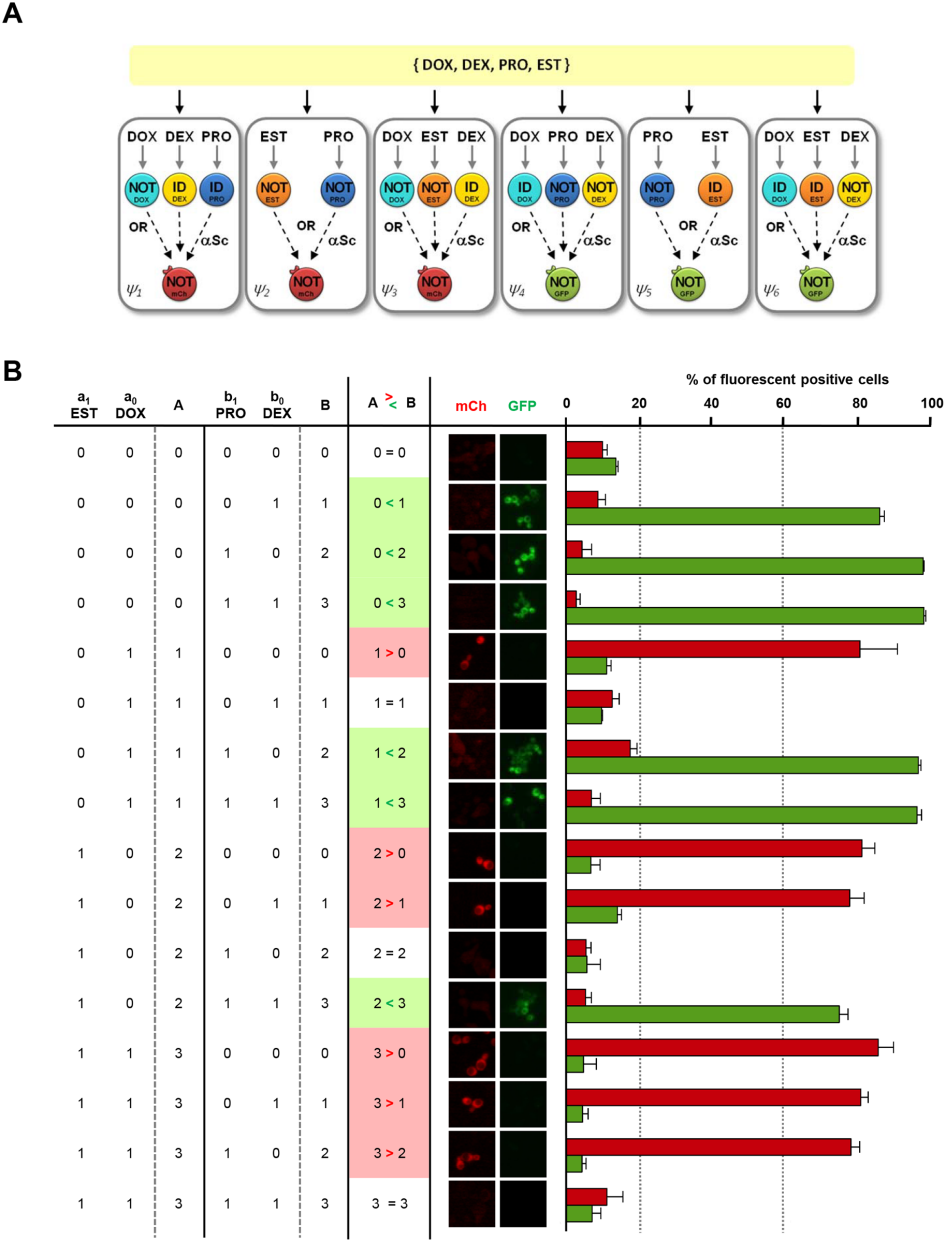
Design and *in vivo* implementation of a 4-input comparator. (A) Schematic representation and spatial distribution of the cells used in the 2 bit magnitude comparator. (B) Truth table (left), microscope images (middle) and percentage of FACS fluorescent-positive cells (right). Cells were mixed proportionally and combinations of four inputs (17-β-estradiol (EST), a_1_; doxycycline (DOX), a_0_; progesterone (PRO), b_1_; and dexamethasone (DEX), b_0_) were added simultaneously to the six chambers. After computing, for each combination of inputs, the percentage of GFP or mCherry positive cells from the corresponding three chambers were analyzed using FACS. Green (GFP) and red (mCherry, (mCh) bars represent output quantification (A>B red; A<B green; A = B no signal). Data represent the mean and standard error of the chamber with highest % of positive cells from three independent experiments.

To explore the potentiality of this approach, we investigated whether more complex devices could be achieved using biosensing devices as reference, since their implementation is simpler in the laboratory when all the input combinations need to be measured. We increased circuit complexity by creating a circuit that responds to four different inputs by producing two different outputs. We chose a 2-bit magnitude comparator, which permits the comparison of two binary numbers, A and B, each having two bits (A = {a_1_, a_0_} and B = {b_1_, b_0_}). Comparators are at the heart of most central processing units (CPUs) in computers and perform a large portion of the logical operations. The circuit is able to respond to 4 inputs, upon 16 entries, and yields three different outcomes from the computation (A>B, A<B and A = B). The formal representation of the circuits is shown in S14C and S14D Fig. The implementation of such a circuit *in vivo* required six different consortia and different combinations of four pairs of IL cells. Cells respond to four stimuli (DOX, EST, PRO and DEX), where EST and DOX encoded A, and PRO and DEX encoded B. Of note, this circuit has an additional level of complexity because it requires two different outputs to distinguish between A<B, A>B and A = B. Therefore, we used two OL cell types, that express green (GFP, OL_1_) or red (mCherry, OL_2_) reporters (Fig 4A). The expected output would be green when A<B, red when A>B and no signal when A = B (Fig 4B). After incubation with the inputs, the fluorescence of the cell consortia was assessed and the final computation was calculated by measuring the percentage of mCherry positive cells present in the first three chambers (A>B) and the percentage of GFP positive cells in the last three chambers (A<B). All 16 combinations yielded the expected outcome, supporting the notion that multiple functions can be constructed from a small library of reusable cells.

**Fig 5:**
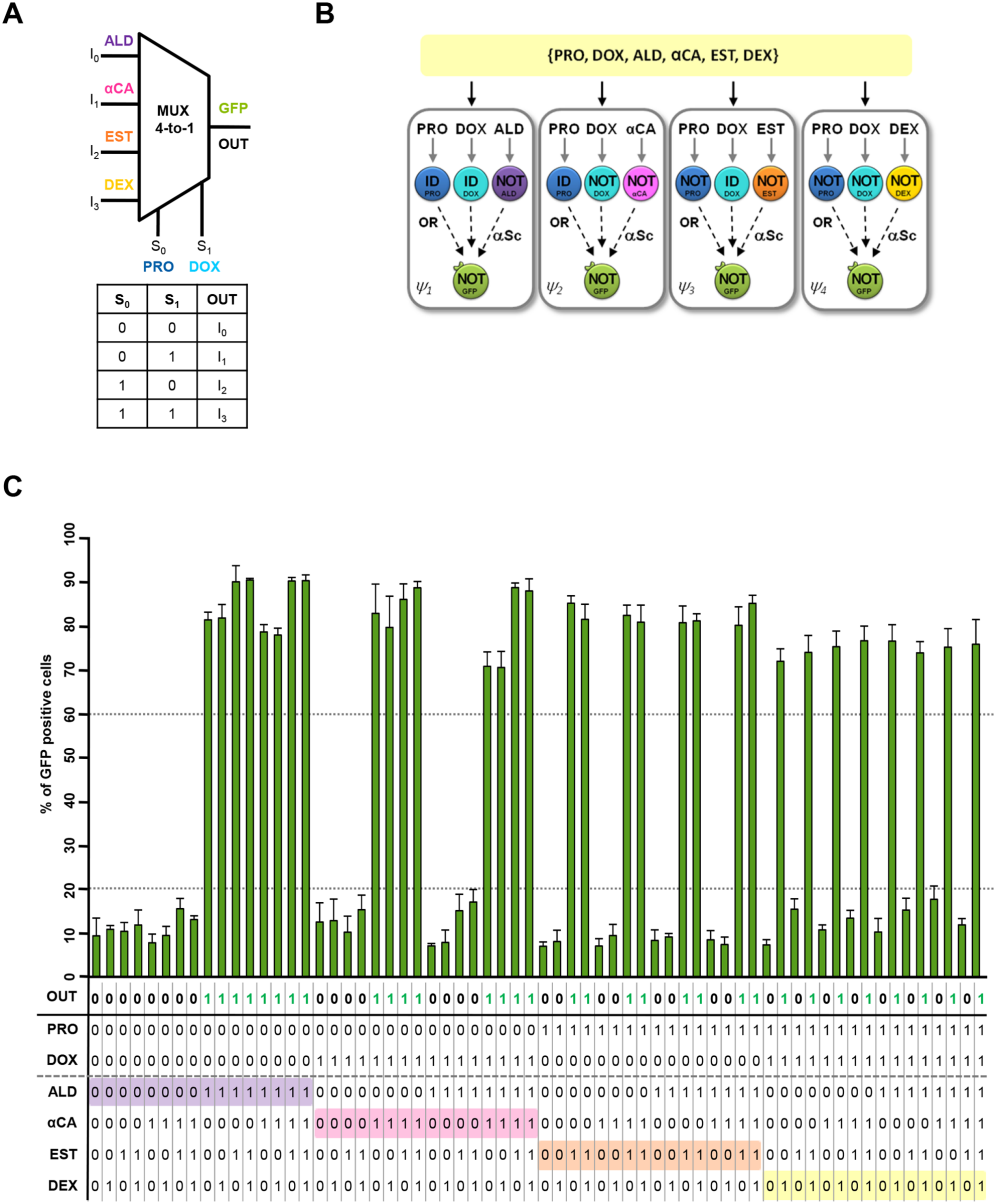
Design and *in vivo* implementation of a 6-input multiplexer (MUX 4-to-1). (A) Schematic representation of the input stimuli (I_0_-I_3_) and selectors (S_0_-S_1_) in the MUX 4-to-1 circuit. (B) Schematic representation and spatial distribution of the cells used in the MUX 4-to-1 circuit. (C) Truth table (bottom) and percentage of GFP-positive cells analyzed by FACS (top). Cells were mixed proportionally and treated with a combination of progesterone (PRO; selector 0, S_0_), doxycycline (DOX; selector 1, S_1_), aldosterone (ALD; input 0, I_0_, violet), *C*. *albicans* α-factor (αCA; input 1, I_1_, pink), 17-β-estradiol (EST; input 2, I_2_, orange), and dexamethasone (DEX; input 3, I_3_, yellow). After computing, for each combination of inputs, the percentage of GFP positive cells from the four chambers was analyzed using FACS. Data represent the mean and standard error of the chamber with highest % of positive cells from three independent experiments.

To demonstrate the scalability of this modular approach and exploit the capability of our library of cells, we implemented a highly complex multiplexer involving 6 inputs. Of note, such computational complexity has never been reached so far in biological circuits. A multiplexer permits the sharing of one device by several signals thereby avoiding the necessity of having one device per input signal. The MUX 4-to-1 is a circuit that responds to 6 inputs. 2 of these 6 inputs are called selectors because they allow the “selection” of which one of the other 4 inputs will determine the final output. Here, PRO and DOX are the selectors (S0-S1) and ALD, αCa, EST and DEX are the inputs (I0-I3). For example, when both PRO and DOX are equal to zero (S0 = 0, S1 = 0), the selected input is ALD (I0) as indicated in the true table (Fig 5A, bottom). Thus, the circuit will produce the 0 output when ALD is equal to 0, and an output of 1 when ALD is equal to 1 (violet row in the truth table, Fig 5C, bottom). Thus, a total of 64 combinations of inputs are possible. The formal representation of the circuits is shown in S14E and S14F Fig. This circuit, which would represent an enormous effort if it was built in a single cell using standard design methods (e.g. S15 Fig), can be assembled by involving just eight IL cells and one OL cell combined in four spatially independent consortia (Fig 5B). Similarly, we directly measured the output from the modules using the fluorescent OL_1_ cells and microscopy and flow cytometry as reader devices. A mixture of the six inputs was simultaneously added to the four chambers and, after incubation, the fluorescence of the consortia was measured using FACS and microscopy. All the 64 combinations of inputs were tested and the final computation was assessed as before. Although the complexity of the circuit required differential outputs to 64 different input combinations, the *in vivo* results clearly showed the expected response (Fig 5C).

## Discussion

A major challenge in the field of synthetic biology is the construction of flexible, scalable and complex logic circuits using engineered cells. Many different strategies have been implemented to create logic circuits in biological systems over the last decade [6]. However, several problems, including those derived from wiring requirements, pose a serious limitation on scalability [6]. Some approaches have been advanced to overcome these obstacles, including the use of multicellular distributed computation [31] and the use of spatially restricted computational modules [32,38,47].

Here, we propose a novel alternative to the standard architecture that combines three elements to create new circuits in a strategic manner: 1) the use of multicellular consortia, 2) spatial segregation and 3) distributed output computation. On top of this, circuit design does not follow electronic standard methodology but rather we implemented a new method that permitted to obtain the maximum benefits of the combination of the three elements (i.e. inverted logic). This approach uses the simplest logic devices, i.e. one-input one-output logic gates, connects the cells of each consortium with a single wire, and allows each consortium to produce the final output independently of the others. This new architecture has several appealing properties. On one hand, using a minimal library of cells, several combinations of multicellular consortia can be assembled (modularity). Modular biocomputing profits from the enormous potential of combining a limited number of building blocks (IL and OL cells), which is comparable with the combinatory richness of standard microelectronics. Once the library of cells has been built, different combinations of the same cells can create novel circuits without additional engineering, thereby pushing the concept of *reusability of parts* one step further. This combination of cells allows an exponential increase in the number of different circuits available without additional engineering.

There are however many aspects that need to be taken into account when designing and implementing logic circuits with biological consortia. An extensive cell characterization is important to create proper cell libraries. It is important that the cells within the same module display similar dynamic responses that can be easily deciphered by the characterization of transfer functions. A possible alternatively, could be to introduce a certain nonlinear circuit such as a toggle in the IL cells [48]. Also, it is essential that each cell responds to only one input and thus crosstalk has to be avoided. The balance of cells within the consortia is also a key point for long term circuit responses. It is critical that no cellular imbalances occur and thus, cellular growth should be similar among cells in a module. After library construction and characterization, that serves to create cells responding to the desired input, logic design of the circuit can be established by defining the best cell combination from the library within the different modules. In general, a given circuit can be implemented by different cells and modules combination, which is optimized *in silico* to reduce the number of cells and modules to facilitate *in vivo* implementation. The reusability of the cells within a library depends on the input that needs to be sensed, however, our data indicates that thanks to the simple logic of each cell (ID and NOT), cells can be created and swapped easily if they maintain similar dynamic responses as described before.

A crucial property of this architecture is that it does not depend on the complexity of the circuit to be implemented, thereby ensuring virtual unlimited scalability, yet maintaining minimal genetic engineering requirements. For instance, a library that responds to six inputs as reported here is sufficient to create up to 1.8x10^19^ different circuits, with a maximum of 32 modules in the worst complex scenario. As a proof of principle, we have built several logic circuits in eukaryotic cells that respond to up to 6 inputs (such as a multiplexer 4-to-1), and that reach an increase in complexity that has not been implemented before. This design shows how a modular biocomputer can be constructed in a flexible, robust and scalable manner. In addition, computation performed by multicellular consortia opens the door to exploration of circuits obtained by combining different cellular species and the synergies that can be derived from this coexistence.

Remarkably, modular biocomputing is flexible to different types of applications; for instance, it can be use to build circuits that function as biosensors and the output of the computation is assessed through a reporter system. These are devices where the circuit outcome can be assessed by microscopy (e.g. microfluidic devices), or biodetectors, analytical and microbiological assays, and diagnostic kits. Still, there are scenarios where the output is a secreted molecule with a biological function, e.g. recombinant proteins or enzymes produced in bioreactors, chemical compounds and metabolites in industrial biotechnology or pharmaceutical products (hormones or drugs) in biomedical applications. By expanding the library with only two simple cells we showed how our design can be extended to such applications as well. Finally, we built an open-flow computing apparatus as a proof of principle of an integrated device that upgrades the potential of our architecture to circuits with a secreted output. Depending on the application, the user may require different devices with different proprieties and a diverse level of spatial isolation within each module. For example, in biotechnological applications, the production of toxic by-products by one cell type in a module may inhibit other cell type, thus affecting the computational capability of the consortia. In such cases, the implementation will require isolation of the different cell types in each module where the toxic product is trapped but the wiring molecule can flow. Also, unbalanced growth rates of different cells types calls for devices where the culture growth can be maintain constant like in a chemostat. All together these concerns are pushing the field towards a personalized device technology where users design and build their own devices specifically optimized for the desired application. Lately, many possible micro-environments, such as microfluidics devices [49–51], cell microcapsules [52], micro-fabricated implantable arrays [53] and cell culture patterns [54] have been improved. Recent advancements in photolithography, plastic molding and, recently, in 3D-printing might lead to custom-designed microdevices easily available for biomedical applications. Coupling these technologies with modular biocomputing design can provide a general and robust way of exploring the landscape of living computational devices.

## Materials and Methods

### Engineered yeast cell library

Yeast W303 (*ade2-1 his3-11*,*15 leu2-3*,*112 trp1-1 ura3-1 can1-100*) cells were genetically modified so that they could produce αSc from an inducible promoter (IL cells), control output expression (fluorescent proteins or αCa) in response to the αSc (OL cells), or produce a fluorescent protein in response to αCa (Buffer cells). Schematic genotypic characteristics of each cell and plasmid used are summarized in S1 Text, S3 and S4 Figs and S1 and S2 Tables. The cells within a consortium can be followed by specific markers or the presence of fluorescent reporters.

### Growth conditions

Overnight cultures were diluted to OD_660 nm_ ≈ 0.2 and were grown at 30°C in YPD or selective medium.

### Characterization of the cellular properties of the engineered cells of the library

We followed standard electronics for defining a positive signal from a circuit as described [31]. As shown in Figs 3–5, in our biological devices the resolution of the 1 logic is more than 60% and 0 logic is less than 20% of the maximal value, indicating that these circuits are comparable with electronics in terms of resolution. However, this separation between logic states is a necessary but not a sufficient condition to guarantee that multicellular circuits can be implemented that connect different cells acting as logic blocks. A proper characterization of the library of engineered cells is necessary to analyze the so-called Transfer Function, i.e. the cellular response with respect to different input levels. An adequate Transfer Function should be characterized by several key features [55,56]: i) a step-like shape, ii) linear or higher gain ranges in order to ensure that the signal will not be degraded from input to output in a single cell, iii) the noise margins must be adequate, without overlap between the high and the low state, and iv) each cell must respond properly only to the specific inputs and must ignore the rest of inputs of the circuit. All these aspects have been experimentally addressed in the set of engineered cells of the library. S6 and S7 Figs show the full set of transfer functions for each cell. Experimental data were fitted to a Hill equation as described in S1 Text and S3 Table. All these curves exhibit the proper shape to be logic blocks for a multicellular implementation. This procedure allows characterization not only of cellular behavior but also of the wire efficiency. Cells were grown in selective media or YPD to mid exponential phase and were then diluted to OD_660 nm_ ≈ 0.2. Input Layer (NOT) cells were washed to remove the αSc that was produced o/n and were resuspended in YPD. Each Input Layer cell was mixed with the GFP Output Layer cell (OL_1_) at a 4:1 ratio and the mixture was subjected to different concentrations of input (S6 Fig). OL_3_ cells were washed to remove the αCa that was produced o/n and were resuspended in YPD. OL_3_ cells were then mixed with the Buffer Layer cells (BL) at a 4:1 ratio and were subjected to different concentrations of αSc (S7A Fig (bottom)). Various concentrations of αSc were added to OL_1_ and OL_2_ cells (S7A Fig (top)) and different concentrations of αCa factor were added to BL cells (S7B Fig). Samples were incubated for 4 h at 30°C and were analyzed using flow cytometry. Data are expressed as the percentage of GFP positive cells. The transfer function represents the mean and standard deviation of three independent experiments. All of the cells exhibit a proper behavior that allows definition of a clear threshold between 0 and 1 logic states. Based on these results, we established the concentration of inputs used in the circuits (arrow in S6 and S7 Figs) to be clearly above the threshold.

### Computational output detection by flow cytometry and microscopy in a single cell

Output of the circuits, transfer function and crosstalk were analyzed after 4 h incubation at 30°C with a combination of inputs unless specified differently. Samples were diluted in PBS and analyzed using flow cytometry (BD LSRFortessa). A total of 10.000 cells were collected from each sample. Constitutive fluorescence in Output Layer cells (mCherry for OL_1_ and YFP for OL_2_), was used to differentiate them from Input Layer cells (S5B and S5D Fig). In S7A Fig, bottom, constitutive fluorescence in the Buffer Layer cells (mCherry) was used to differentiate them from the OL_3_ cells. Specific emission in the fluorescence channel of the subsets of Output or Buffer Layer cells was measured versus autofluorescence (PerCP-Cy5-5-A channel for GFP and YFP, PerCP-Cy7 channel for mCherry). Autofluorescence in a wild type strain without carrying any reporter was measured as a reference (S5A Fig). A gate was set to subtract autofluorescence and cells inside the gate were considered as GFP positive cells. Data are expressed as percentage of fluorescent positive cells (GFP for OL_1_ and BL, mCherry for OL_2_) (S5C and S5E Fig). Also measured in a shift on total fluorescence (S5F Fig). An output expression below the 20% of GFP positive cells corresponded to the 0 logic state (low threshold) and above the 60% of GFP positive cells corresponded to the 1 logic state (high threshold). In all the circuits, we use the same low and high threshold values. Data were analyzed using FlowJo or BD FACSDiva software. A representative FACS plot of our quantification method is presented in S5 and S11 Figs. For microscopic analyses, cells were harvested and resuspended in Low Fluorescent Media. Images were collected with a Nikon Eclipse Ti Microscope using NIS elements Software (Nikon) and were analyzed using ImageJ.

### Crosstalk analyses

Fig 2D shows the individual cellular response of each IL cell in response to the different single inputs they encounter within a circuit or to all of the inputs combined. Cells were grown in selective media or YPD to mid exponential phase (OD_660 nm_ ≈ 0.2). Input Layer (ID) cells were mixed with the GFP Output Layer cells at a 2:1 ratio. Input Layer (NOT) cells were washed to remove the *S*. *cerevisiae* alpha factor that was produced o/n, were resuspended in YPD and were mixed with the GFP Output Layer cells at a 3:1 ratio. Each mixture was subjected to all 6 inputs individually, to all 6 inputs together (ALL) and to all inputs except for the specific input (ALL-I). Samples were incubated for 4 h at 30°C and were analyzed using flow cytometry. Data are expressed as the percentage of GFP positive cells. The experimental data shows that there is no undesired crosstalk and that each cell responds only to the expected input.

### Construction of an open flow computational device

The open flow device (Fig 3B) is composed of three parts: the *computational* chambers (*ψ*_*1*_, *ψ*_*2*_, *ψ*_*3*_), the valves, and the *Buffer* chamber (BUF). The *computational* chambers are tanks with 4.5ml liquid storage and a cup allowing pneumatic actuation of the fluids (Microfluidic ChipShop). 1.6 mm tygon tubes connect the air pump (CellASIC ONIX Control System) with the tanks cup using male mini-luer connectors. The fluidic interface is realized as female luer connector. Valves (Discofix Braun) can be turned in three different positions (*p*_*1*_: waste, *p*_*2*_: closed and *p*_*3*_: Buffer) according to the different experimental steps. The *Buffer* chamber is a 4.5ml tank with a pneumatic cap carrying three male mini luers. The interconnection between the components is enables by 1.6 mm tygon tubes, male and female luers and mini luers. To prevent the cells mixture to enter the OR chamber, but still allowing the transferring of the supernatant, a 0.22 mm Millipore filter is plugged in before the OR chamber. Finally, a device carrier has been designed and built to hold the apparatus.

### Buffer Layer cells experiment

Fig 2B shows the single cell computation in the presence of the optional Buffer Layer cells. Input Layer cells were mixed with OL_3_ cells in the absence or presence of the specific input. After 4 h of computation the supernatant of the mix was added to the Buffer Layer cells, incubated for 4 h and the percentage of GFP positive BL cells was analyzed using FACS. Fig 3B and 3C show the implementation of the major rule circuit using the optional BL and the open flow device. The appropriate combinations of IL cells were mixed proportionally into the three chambers, together with the OL_3_ cells, and exposed to the same combination of the three inputs. After 7h of incubation stirring at RT (*transitory time*), we pumped into the chambers fresh media with the corresponding combination of inputs (psi: 0.5, valve: *p*_*1*_, minutes: 3). The valve was then turned to *p*_*2*_ and the cells mixture was incubated for 10 h stirring at RT (*computational time*). Finally, the αCa produced by the independent modules was automatically collected in the *Buffer* chamber (psi: 5, valve: *p*_*3*_, minutes: 1) and incubated with the BL cells for 4 h at 30°C. Samples were analyzed using FACS and microscopy. We repeated the same experiment in triplicate for each combination of inputs of the majority rule.

### Implementation of transducer circuits

Circuits in Figs 3–5 were built mixing proportionally the appropriate combination of IL and OL cells in different tubes (i.e. the consortia). The same mixture of inputs was simultaneously added to each consortium. All the possible combinations of inputs were tested. After 4h of computation at 30°C, for each combination of inputs, the percentage of GFP positive cells in each module was analyzed using FACS and microscope. A positive signal (more than 60%) in any consortia accounts for a 1 as the final output of the circuit. When more than one consortium gave a positive fluorescent signal we choose the highest value. The same was done for negative (less than 20%) outputs (0) (S11 Fig). Data represent the mean and standard error of three independent experiments.

## Supporting Information

S1 Text Supporting information including: Design of minimal logic circuits based on inverted logic formulation (ILF), transfer function fitting, fluorescence data analysis, full description of each cell used in the biological circuits and Engineered Input Layer cells that respond to hormones. (DOCX)

S1 Table Yeast strains used in this study. (DOCX)

S2 Table Plasmids strains used in this study. (DOCX)

S3 Table Fitting parameters and correlation coefficient *r*. (DOCX)

S1 Fig Relationship between the number of implementable functions and the number of cells and modules required. (**A, B, C**) Dependence of the number of possible implementable functions with respect to the number of different cells required. Data for functions with 2, 3 and 4 inputs are shown. (**D, E, F**) Dependence of the number of possible implementable functions with respect to the number of different modules (consortia) required. Data for functions with 2, 3 and 4 inputs are shown.
(TIF)

S2 Fig Graphic representation of the architecture of the Input, Output and Buffer Layers cells. (**A**) Identity cells (ID; top) express the *S*. *cerevisiae* alpha factor pheromone under the control of an input-inducible promoter. NOT cells (NOT; bottom) constitutively express this pheromone under the control of a modified *TEF1* promoter (*TEF1-OplacI*). In the presence of input, *S*. *cerevisiae* alpha factor expression is repressed by LacI. (**B**) An ssrA-tagged version of yEGFP was expressed under the control of the *TEF1i* promoter. The ssrA tag allows the Clp protease complex to recognize yEGFP^srrA^ and induce its degradation. The protease subunit ClpP is constitutively transcribed under the *ADH1* promoter, whereas the ClpX subunit and the LacI repressor were expressed under the control of the *FUS1* promoter, which is induced by *S*. *cerevisiae* alpha factor. (**C**) OL_3_ cells constitutively express the *C*. *albicans* alpha pheromone under the control of a modified *TEF1* promoter (*TEF1-OplacI*). The LacI repressor was expressed under the control of the *FUS1* promoter, which is induced by *S*. *cerevisiae* alpha factor. (**D**) Buffer Layer (BL) cells express the *C*. *albicans* pheromone receptor *STE2* and produce GFP in the presence of *C*. *albicans* alpha factor.
(TIF)

S3 Fig Schematic representation and basic genetic information of the Input Layer cells. Cells in the library respond to six different inputs (DOX; doxycycline, PRO; progesterone, ALD; aldosterone, αCa; *C*. *albicans* alpha factor, EST; 17-β-estradiol, DEX; dexamethasone) with two different logics. In the presence of the input, Identity cells (ID, left) express *S*. *cerevisiae* alpha factor, whereas NOT cells (NOT, right) repress pheromone production in response to stimuli. All cells are W303 derivatives.
(TIF)

S4 Fig Schematic representation and basic genetic information of the Output Layer and Buffer Layer cells. (**A**) The Output Layer cells sense *S*. *cerevisiae* alpha factor and shut down the expression of a fluorescent protein (GFP, mCherry) or the production of *C*. *albicans* alpha factor. All cells are W303 derivatives. (**B**) The Buffer Layer cell sense *C*. *albicans* alpha factor and produce GFP. Cell is W303 derivative.
(TIF)

S5 Fig Representative FACS analysis using quantitative single cell output. Fluorescence from Output Layer and Buffer Layer cells was assessed by flow cytometry. A total of 10.000 cells were analyzed. (A) Representative FACS plot of a wild type W303 cells. (**B**) Panel shows mCherry intensity (Y axis) *versus* autofluorescence (X axis) and allows selecting the OL_1_, or BL, cells (mCherry positive) from the Input Layer cells (mCherry negative). (**C**) Selected OL_1_, or BL, mCherry cells were analyzed by their GFP expression (Y axis) *versus* autofluorescence (X axis). Two examples are given: a GFP positive sample (left) and GFP negative one (right). (**D**) OL_2_ cells are analyzed as in A, using the YFP channel to select them from the Input Layer cells. (**E**) Selected YFP cells were assessed by their mCherry expression. Two examples are given: a mCherry positive sample (left) and mCherry negative one (right). (F) Population density and histograms plots of fluorescence intensities of OL_1_, OL_2_ and BUF cells. Histograms plots are compared to density plots in presence or absence of the corresponding alpha factor.
(TIF)

S6 Fig Transfer Function analyses of the Input Layer cell library. Input Layer cells were mixed with the Output Layer GFP cells (OL_1_) and treated with different inputs concentrations. Samples were incubated for 4h at 30°C and analyzed by FACS. Data are expressed as the percentage of GFP positive cells and represent the mean and standard deviation of three independent experiments. Arrows indicate the working concentrations of inputs.
(TIF)

S7 Fig Transfer Function analyses of the Output Layer and Buffer Layer cells. (**A**) Output Layer cells OL_1_ (upper left) and OL_2_ (upper right) were incubated with different concentrations of *S*. *cerevisiae* alpha-factor and analyzed as in S6 Fig. Output Layer cells OL_3_ (lower left) were incubated with Buffer Layer cells in the presence of different concentrations of *S*. *cerevisiae* alpha-factor and analyzed as in S6 Fig. (**B**) Buffer Layer cells were incubated with different concentrations of *C*. *albicans* alpha factor and analyzed as in S6 Fig.
(TIF)

S8 Fig Growth curve of the Input Layer cells library. Exponential cultures of Input Layer cells were diluted to OD_660 nm_ ≈ 0.02 and their growth curve was measured using Synergy H1 BioTeK for 24 h. Data represent the mean and standard deviation of three independent experiments.
(TIF)

S9 Fig Growth curve of the Output Layer and Buffer Layer cells. (**A**) Output Layer cells OL_1_ (upper left), OL_2_ (upper right) and OL_3_ (lower left) growth curve was measured as in S8 Fig. (**B**) Buffer Layer cells growth curve was measured as in S8 Fig.
(TIF)

S10 Fig Examples of 2-inputs logic gates implemented with the library of cells. (A) AND gate. (B) NOR gate. C) N-IMPLIES gate. Schematic representation of the cells used in the circuits (left). Truth table (middle). Percentage of OL_1_ GFP-positive cells (right). Cells were mixed proportionally and treated with different combinations of inputs. After computing, for each combination of inputs, the percentage of OL_1_ GFP positive cells was analyzed using FACS. Data were analyzed and processed as described in Material and Methods and represent the mean and standard error of three independent experiments.
(TIFF)

S11 Fig Example of computational output detection and data quantification. (**A**) The quantification of one majority rule experiment is showed as an illustrative example of all circuits’ quantification and data treatment. For every chamber, and for each combination of inputs, fluorescence intensity of the subsets of OL cells was measured versus autofluorescence. Data are expressed as percentage of GFP positive cells and analyzed using FlowJo. (**B**) When more than one consortium gave a positive fluorescent signal we choose the highest value as the final circuit’s output.
(TIF)

S12 Fig GFP data analysis. (**A**) Transfer function data of the OL_1_ cells expressed as GFP a.u. Values ranges from maximum of 1600 to a minimum of 450 GFP a.u and the curve presents a step like shape. Experimental transfer function data were fitted to a Hill equation as described in S1 Text. (**B**) Normalized GFP (a.u.) transfer function of OL_1_ cells (round circles) overlapped with the same transfer function where data are expressed as % of GFP positive cells (diamonds). Experimental transfer functions data were fitted to a Hill equation as described in S1 Text (GFP a.u., dotted line; % GFP, straight line). Both transfer functions exhibit a proper behavior that allows the definition of a clear threshold between 0 and 1 logic states. (**C**) Schematic representation and spatial distribution of the cells used in the majority rule circuit (left). Truth table (middle). Results of the majority rule circuit presented in Fig 3C, green bars, analyzed as normalized GFP (a.u.) (right). Data represent the mean and standard error of three independent experiments.
(TIF)

S13 Fig Design and *in vivo* implementation of a 3-input majority rule. (**A**) Schematic representation and spatial distribution of the cells used in the majority rule circuit. The circuit is the same as in Fig 3 except here we used IL_7_ cells, which respond to DOX (right), instead of the previously used IL_12_ cells, which respond to DEX (left). (**B**) Truth table (left) and percentage of FACS GFP positive cells (right). Yellow bars refer to the circuit built with IL_12_ (DEX) visualized also in Fig 3C, green; cyan refers to the same circuit built with IL_7_ (DOX). Data represent the mean and standard error of three independent experiments.
(TIF)

S14 Fig Logic representation of the circuits implemented according to the standard methodology or the novel approach based on ILF. Despite the logic representation of circuits based on ILF involves NOT and OR logic gates, only NOT gates are genetically implemented. OR logic is implicitly implemented by spatial segregation of the consortia. In each consortium, the IL cells produce the same wire molecule in a shared environment thus implementing an implicit OR logic gate. Combining this OR gate with the NOT gate of the OL cells results in a multi-input NOR gate. (**A**) ILF design of the majority rule circuit. (**B**) Standard design of the majority rule circuit. (**C**) ILF design of the 2-bit magnitude comparator. (**D**) Standard design of the 2-bit magnitude comparator. (**E**) ILF design of the multiplexer MUX4to1. (**F**) Standard design of the multiplexer MUX4to1.
(TIF)

S15 Fig Theoretical implementation in single cell of a MUX4to1 multiplexer circuit according to standard methodology for circuit design. This circuit requires 10 different promoters, 6 regulated by the external inputs A, B, C, D, E, and F, and 4 for internal connections. Additionally, 8 different wires are necessary (dashed lines), implemented by 8 different repressor proteins.
(TIF)
