# Supplementary material for: Implementation of Complex Biological Logic Circuits Using Spatially Distributed Multicellular Consortia
Source: PLoS Comput Biol. 2016 Feb 1;12(2):e1004685. doi: 10.1371/journal.pcbi.1004685 (PMC4734778; doi:10.1371/journal.pcbi.1004685)
Supplement: S1 Text — (DOCX) [file pcbi.1004685.s001.docx]

**Supporting Text S1**

**Design of minimal logic circuits based on inverted logic formulation (ILF)**

Inverted Logic Formulation (ILF)

Multicellular implementation of ILF with spatial segregation

Spatial segregation as an additional computational element

Example: the Majority Rule Circuit

Circuits’ scalability

**Transfer function fitting**

**Fluorescence data analysis**

**Full description of each cell used in the biological circuits**

**Engineered Input Layer cells that respond to hormones**

**Design of minimal logic circuits based on inverted logic formulation (ILF):**

Despite the enormous efforts devoted to developing complex logic circuits able to perform non-trivial decision-making processes, the results obtained are far from the expected ones. Current limitations do not allow the degree of complexity necessary for industrial, environmental, or biomedical applications [1]. Logic circuits are made up from basic logic gates that, connected together, constitute the building blocks to produce more complex circuits. Several aspects determine the complexity, and hence the limitations, of a circuit, namely i) the complexity of the building blocks, e.g. logic gates, ii) the pattern of connections and, iii) the number of different wires involved.

Any set of logic gates which can be used to implement all possible logic functions only combining members of a given set is called *functional complete set*. Not all logic gates are equally complex. For instance, biological implementation of the OR logic is easier than other logics, such as AND or XOR logics [2,3]. Additionally, logic gates that respond to a single input (Identity and NOT gates) are easier to implement than two-inputs (or higher) logic gates. Based on these arguments, the easier biological implementation of complex logic circuits can be achieved by combining several one-input logic gates with an OR logic, and by using the minimal number of wires with the simplest pattern of connections. Of note, the set {OR, NOT} defines a *functional complete set* [4].

**Inverted Logic Formulation (ILF).** To analyze how a logic function can result in the optimal circuit implementation, we started with the formal definition of a logic function involving N inputs and one output. A logic function can be described by the so-called truth table, where all possible combinations of inputs and the associated outputs are defined. Systematically applying Boolean algebra rules [5], a given function can be expressed as a minimal combination of NOT, OR, and AND binary operators in the canonical form. Although there are different but equivalent expressions of a Boolean function, here we focused on minterms [6]. By this formalism, any arbitrary Boolean function with N inputs has the following general expression:

$$\begin{matrix} f=[\phi_{11}(x_{1})AND \phi_{12}(x_{2})\cdot\cdot\cdot AND \phi_{1N}(x_{N})]OR[\phi_{21}(x_{1})AND \\ \phi_{22}(x_{2})\cdot\cdot\cdot AND \phi_{2N}(x_{N})]OR\cdot\cdot\cdot[\phi_{M1}(x_{1})AND\phi_{M2}(x_{2})\cdot\cdot\cdot AND\phi_{MN}(x_{N})] \end{matrix}$$

or in a compact form:

In which Σ represents the OR operator and Π the AND operator. The function *φ_ij_* is either a logic representation of the presence of a molecular input *x_j_* (Identity function) or of its absence (NOT function). Finally, *M* is the maximum number of terms present in the Boolean function, which depends on the complexity of the function, but the condition *M* ≤ 2^N-1^ is always satisfied [5]. The expression of a Boolean function *f* can be reduced by the systematic application of standard rules of simplification, such as the so-called Karnaugh maps [7] or the Quine-McCluskey algorithm [8].

A simplified expression of the canonical form is not the minimal implementation possible in a cellular context. Easier implementations can be systematically achieved modifying the canonical expression of the Boolean function to obtain an expression involving only OR logic (the simpler logic in a cellular implementation). This goal can be achieved applying a double negation, i.e.

According to Morgan’s Laws [5],

$$\left\{\begin{matrix} \overline{aORb}=\overline{a}AND\overline{b} \\ \overline{aANDb}=\overline{a}OR\overline{b} \end{matrix}\right.$$

the Boolean function can be expressed as:

where .

Hence, the Boolean function results in the OR combination of several computational modules *ψ_i_*, i.e.: $f=\sum_{i=1}^{M} \psi_{i}$

These modules *ψ_i_* are the inversion of OR combinations (symbol Σ) of inverted terms *θ_ij_*, what we call *Inverted Logic Formulation* (ILF), i.e.

$$\psi_{i}=\overline{\sum_{i=1}^{N} \theta_{ij}(x_{j})}$$

Functions *θ_ij_(x_j_)* can be chosen among NOT or Identity functions, i.e.

$$\theta_{ij}(x_{j})=\left\{\begin{matrix} \overline{\begin{matrix} x_{j} \\ or \end{matrix}} \\ x_{j} \end{matrix}\right.$$

depending on the specific function to be implemented by the circuit.

**Multicellular implementation of ILF with spatial segregation.** Using ILF, the Boolean function now is expressed as an OR combination of different computational modules *ψ_i_*, and can be systematically translated into cellular circuits. The biological implementation is simplified by two means. First, the circuit is distributed into several cellular types (distributed computation [9,10]). Second, each *ψ_i_* modules can produce the output, the so-called distributed output [9,10]. An example could be a secretable molecule, e.g. hormones, which will give an output of 1 no matter by which *ψ_i_* modules is produced.

In this embodiment, each module *ψ_i_* can be organized in two cell layers. The first one is the *Input Layer* made by cells that sense external inputs *x_j_*. and implement a one-input one-output logic function *θ_ij_*(*x_j_*) (Identity or NOT). Every IL cell, in response to the external input *x_j_*, secretes or not a wiring molecule *ω*. The wiring molecule is the same for each cell; hence, once it is secreted into the medium and mixed, the OR logic between the different functions *θ_ij_*(*x_j_*) into the same *ψ_i_* module is performed.

The second layer of cells (*Output Layer*) is composed by a single cell type that implements a NOT function: in presence of the secreted wiring molecule, the output is not expressed whereas in its absence, the final output is expressed. Thus, the final response of *ψ_i_* is inverted. Fig. 1A shows a schematic diagram of the architecture of a computational module *ψ_i_*.

**Spatial segregation as an additional computational element.** The global function *f* is the OR combination of different modules *ψ_i_*. Here, the distributed output leads to the direct implementation of the OR function by default [10] and no additional elements are required. Still, for the correct computation, no cross-talks between different *ψ_i_* are allowed. The number *M* of required computational modules *ψ_i_* depends on the complexity of the Boolean function *f*, as well as the number of different wiring molecules needed. We introduced spatial segregation of the cells as a new computational element. If each *ψ_i_* remains physically isolated, the same wiring molecule can be used in all modules, and the so-called wiring problem, in which every wire needs to be a different chemical entity, is now reduced since only one wire is needed for any given circuit independently on its complexity. The computational complexity of the function is encoded by the number of different chambers involved. For instance, we can consider a device in which each module *ψ_i_* is located in a different chamber (Fig. 3B). Alternatively, other systems allowing physical separation of cells such as microfluidics and microencapsulation are suitable to be considered for the implementation of these types of circuits.

**Example: the Majority Rule Circuit.** The experimental procedure to determine the best consortia combination is based on design and testing. Circuit design is first done *in silico* following ILF systematic methodology for logic circuit implementation which ensures the use of the correct combination of cells. For illustrative purposes, we applied the ILF to design a majority rule circuit. Majority rule circuits are a decision-making systems based on the presence of more than the half of all possible inputs. These types of circuits are typically used in electronics as security devices. In a biological context, these types of decision-making circuits can be used, for instance, to determine the existence of the minimal conditions to trigger a cellular process. The truth table describing the behavior of a majority rule circuit is the following:

| *x_1_* | *x_2_* | *x_3_* | *f* |
| --- | --- | --- | --- |
| 0 | 0 | 0 | 0 |
| 0 | 0 | 1 | 0 |
| 0 | 1 | 0 | 0 |
| 0 | 1 | 1 | 1 |
| 1 | 0 | 0 | 0 |
| 1 | 0 | 1 | 1 |
| 1 | 1 | 0 | 1 |
| 1 | 1 | 1 | 1 |

The corresponding canonical form is:

Here functions *φ_11_*, *φ_22_*, *φ_33_* are NOT functions, whereas the rest are Identity functions, i.e.

$$\begin{matrix} f=\left[\overline{x_{1}}ANDx_{2}ANDx_{3}\right]OR\left[x_{1}AND\overline{x_{2}}ANDx_{3}\right] \\ OR\left[x_{1}ANDx_{2}AND\overline{x_{3}}\right]OR\left[x_{1}ANDx_{2}ANDx_{3}\right] \end{matrix}$$

After applying a simplification method, e.g. Karnaugh maps, this function can be reduced to:

$$f=\left[x_{1}ANDx_{2}\right]OR\left[x_{1}ANDx_{3}\right]OR\left[x_{2}ANDx_{3}\right]$$

Starting from the standard implementation of this circuit, made combining OR and AND logic gates,, it is possible to reduce the complexity of the circuit by i) simplifying the logic gates involved, and ii) reducing the number of wires by applying the method presented above, i.e.:

$$\begin{matrix} f=\overline{f}=\overline{\left[x_{1}ANDx_{2}\right]OR\left[x_{1}ANDx_{3}\right]OR\left[x_{2}ANDx_{3}\right]} \\ \\ f=\overline{\overline{\left[x_{1}ANDx_{2}\right]}AND \overline{\left[x_{1}ANDx_{3}\right]}AND\overline{\left[x_{2}ANDx_{3}\right]}} \\ \\ f=\overline{\left[\overline{x_{1}}OR\overline{x_{2}}\right]AND\left[\overline{x_{1}}OR\overline{x_{3}}\right]AND\left[\overline{x_{2}}OR\overline{x_{3}}\right]} \\ \\ f=\overline{\left[\overline{x_{1}}OR\overline{x_{2}}\right]}OR\overline{\left[\overline{x_{1}}OR\overline{x_{3}}\right]}OR\overline{\left[\overline{x_{2}}OR\overline{x_{3}}\right]} \end{matrix}$$

Therefore, in this example the circuit can be implemented by using the OR combination of three computational modules, i.e.

$$f=\psi_{1}OR\psi_{2}OR\psi_{3}$$

with

$$\begin{matrix} \psi_{1}=\overline{\theta_{11}(x_{1})OR\theta_{12}(x_{2})}=\overline{\overline{x_{1}}OR\overline{x_{2}}} \\ \psi_{2}=\overline{\theta_{21}(x_{1})OR\theta_{23}(x_{3})}=\overline{\overline{x_{1}}OR\overline{x_{3}}} \\ \psi_{3}=\overline{\theta_{32}(x_{2})OR\theta_{33}(x_{3})}=\overline{\overline{x_{2}}OR\overline{x_{3}}} \end{matrix}$$

In a spatially segregated embodiment, this circuit requires three different chambers (or microcapsules), one for each *ψ_i_*. The first chamber will contain two cells in the Input Layer. The first cell will respond to input *x_1_* producing the wiring molecule *ω* according to the NOT logic. The second cell will sense the input *x_2_* and produce the same *ω* molecule, following the same NOT logic. Finally, a single cell type that will produce the final output in absence of the *ω* molecule forms the Output Layer. The rest of the chambers have the same architecture, differing in the Input Layer cell types but using the same *ω* molecule and the same cell type in the Output Layer. Of note, the same cell type can be used in different chambers. For instance, the same Input Layer cell responding to *x_1_* is present simultaneously in chambers *ψ*_1_ and *ψ*_2_.

**Circuits’ scalability.** According to the previous results, the requirements for the implementation of any arbitrary complex Boolean function *f* involving *N* different inputs are:

1. A library of engineered cell types that in response to a single input *x_j_* (Input Layer) secrete a molecule *ω* according to either the Identity or the NOT logic, i.e. *ω* is expressed in presence of *x_j_* (Identity) or *ω* is expressed in absence of *x_j_* (NOT). For a function of N inputs, 2·N different cell types must be engineered (N Identities and N NOTs).
2. A single cell type that in absence of *ω* expresses the final output (inverted logic of the Output Layer).
3. Several physically isolated chambers or capsules to allocate the different computational modules (Input and Output Layers). The number of chambers depends on the circuit complexity. Still, this number will be lower than 2^N-1^ which is the upper bound for the number of terms of the simplest canonical form of a Boolean function [5].

One method to demonstrate that the number of modules correspond to 2^N-1^ is based on the Karnaugh map simplification. Karnaugh map is a graphic method to represent a true table. It consists on a lattice formed by 2^N squares where each square corresponds to each possible input combination in the true table. In the squares corresponding to an input combination associated to output 1 we write 1, whereas in the rest we write 0. The following figure illustrates this method:


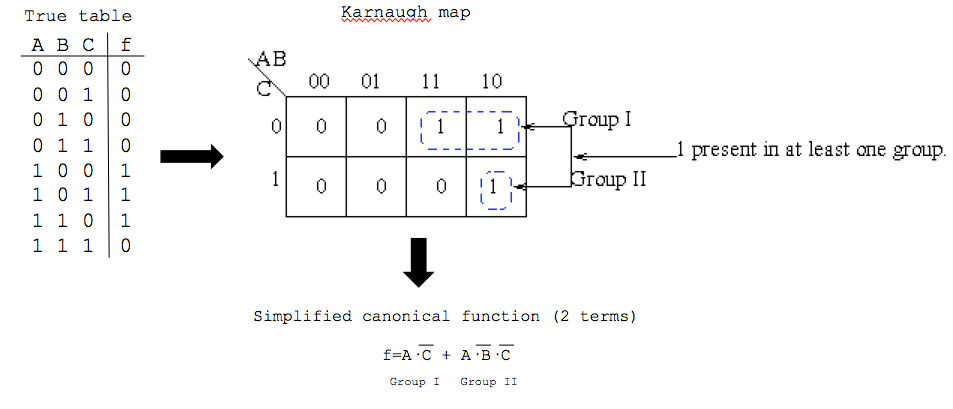


The simplification rules are simple since groups of adjacent squares can be formed having 1 inside. These groups must contain 1, 2, 4 or 8 squares. If a square containing 1 is not grouped with other squares it becomes a group by itself. The number of terms present in the canonical form of the Boolean function will be the same as the number of groups in the Karnaugh map. Each term of the canonical Boolean function will correspond to a module (consortia) in the biological implementation.

From this, it can be concluded that the biggest function (involving the maximum number of groups) corresponds to the case where there are not adjacent squares containing 1. Geometrically this case is a map with alternation between 0 and 1, as the example shown in the next figure. These cases involve 2^(N-1) groups (formed by a single 1 each group), which is the maximum number of different consortia in the biological implementation. We thank the reviewer for asking to clarify this point since this is a very important characteristic of our system that serves to reduce the number of consortia needed to implement a given circuit.


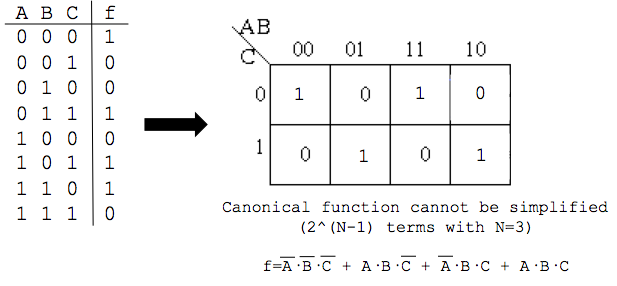


Taking all those elements into consideration, the number of different Boolean functions that can be implemented increases as$2^{2^{N}}$, by using a library of 2·N+1 cells. Fig. 1C compares, for different number of inputs N, the scalability of the upper bound requirements with the number of implementable functions. Fig. S1 shows a detailed analysis of the dependence between number of cells (A-C) and modules (D-F) with respect to the number of functions that can be implemented. Of note, adding a new cell type significantly increase the number of functions. For instance, for functions with four inputs increasing the number of cells from 7 to 8 allows implementing around 40.000 new functions.

**Transfer function fitting:**

Experimental transfer functions data were fitted to a Hill equation according to:

$$T_{f}=\gamma\left(\frac{1+\alpha\cdot\omega\cdot I^{n}}{1+\omega\cdot I^{n}}\right)$$

Fitting parameters and correlation coefficient *r* are listed in the Table S3.

**Fluorescence data analysis:**

Output of the circuits, transfer function and crosstalk were analyzed following the same procedure. Samples were diluted in PBS and analyzed using flow cytometry (BD LSRFortessa^TM^), with the same experimental set up. GFP and YFP fluorescence’s were quantified exiting at 488 nm and mCherry at 561 nm. Constitutive fluorescence in Output Layer cells (mCherry for OL_1_ and YFP for OL_2_), was used to differentiate them from Input Layer cells (Fig. S5B and E). In Fig. S7A, bottom, constitutive fluorescence in the Buffer Layer cells (mCherry) was used to differentiate them from the OL_3_ cells. Data were also treated identically: specific emission in the fluorescence channel of the subsets of Output or Buffer Layer cells was measured versus autofluorescence (PerCP-Cy5-5-A channel for GFP and YFP, PerCP-Cy7 channel for mCherry). A gate was set to subtract autofluorescence and cells inside the gate were considered as fluorescent positive cells. Data are expressed as percentage of fluorescent positive cells (GFP for OL_1_ and BL, mCherry for OL_2_) (Fig. S5C and E). An output expression below the 20% of fluorescent positive cells corresponded to the 0 logic state (low threshold) and above the 60% of fluorescent positive cells corresponded to the 1 logic state (high threshold). In all the circuits, we use the same low and high threshold values. GFP data were also analyzed as GFP mean intensity per cell (GFP a.u.) to check if there was a direct correlation between the % of GFP positive cells (% GFP) and the total amount of fluorescence in a.u. (Fig. S12A-C). Transfer function data of the OL_1_ cells as GFP a.u. was compared with an output expressed as % GFP (Fig. S12 A and B). We choose the OL_1_ cells because it is the main output reporter we used in all transducer circuits (Figs. 3-5). Of note, analyzing the transfer function upon addiction of different synthetic αF concentrations, we can have a clearer idea of the maximum, intermediate and minimum level of fluorescence intensity we are dealing with using this reporter system. As describes in Fig. S12A, these values range approximately from a maximum of 1600 to a minimum of 450 GFP a.u. Also, the transfer function expressed as GFP a.u. presents a step like shape, where the ON state is clearly different from the OFF state. When normalized to 100 the GFP a.u. transfer function clearly overlaps to the transfer function calculated in % of GFP positive cells (Fig. S12B). Both transfer functions display a similar cell response that allows definition of a clear threshold between 0 and 1 logic states. These data indicate that, in this particular type of circuits, we can use the two metrics indiscriminately, because there is a direct correlation between the % of positive cells and the total amount of fluorescence in a.u. To confirm the validity of these conclusions, we analyzed as GFP a.u. the results of the majority rule circuit presented in Fig. 3A, right. We found that the results were consistent with the results presented in % of GFP positive cells (Fig. S12C). Therefore we can conclude that in this type of circuits, output can be calculated using both metrics with similar results. Data were analyzed using FlowJo or BD FACSDiva software.

**Full description of each cell used in the biological circuits:**

To set up the logic circuits we constructed a library of cells that respond to a number of inputs. Before setting up the logic circuits each cell was characterized to prevent differences in output production and cellular growth. Briefly, once an IL cell is engineered, this is coupled to an OL cell in the presence or in the absence of the input to check that the wire connection works properly, the output signal is strong and that showed a clear separation between 0 and 1 logic states. These analyses also serve to compare the different cell responses. When a certain cell does not perform properly, then it is either discarded or optimized.

**Input Layer (IL) cells:** IL cells were engineered in pairs; each pair of cells contains a cell that secretes *S. cerevisiae* alpha factor (αSc) in the presence of a given input (ID logic) and a second cell that secretes αSc in the absence of the same input (NOT logic). IL cells are *MAT*α yeast cells in which *MFα1* and *MFα2* genes were deleted to avoid endogenous alpha factor expression. The *STE3* receptor was also deleted to prevent mating with *MAT*a cells within the circuit. Except for the TetON-TetOFF system, which performs the ID-NOT logic in response to doxycycline (DOX), all the other pairs of cells share the same internal genetic architecture. In the ID cells, wiring molecule (αSc) expression is controlled by a specific input inducible promoter (e.g. the *GAL1* promoter that responds to progesterone (PRO) to induce the *MFα*1 gene) (Fig. S2A, top). In NOT cells, the αSc is constitutively expressed under the control of the engineered promoter *TEF1i* that contains a LacI binding site *(P_TEF1_-OplacI-MFα1)*, and the LacI repressor is transcribed from the same specific input inducible promoter (e.g. the *GAL1* promoter that responds to PRO to induce the repressor of *MFα1* gene expression) (Fig. S2A, bottom). Cells in the library respond to six different inputs: DOX (0.5 µg/mL), PRO (130 nM), aldosterone (ALD) (20 µM), *C. albicans* alpha factor (αCa) (1 µM), dexamethasone (DEX) (128 µM) and 17-β-estradiol (EST) (20 nM).

**Cell#IL1 (ID DOX)** is a cell that produces αSc when DOX is present in the media. IL_1_ cells express the *MFα1* gene under the control of two TetOperators in the centromeric plasmid *YCpTetO_2_-MFα1* that also constitutively expresses the reverse Tet Transactivator (rtTA).

**Cell#IL2 (ID PRO)** is a cell that produces αSc in the presence of PRO. This cell contains the episomal plasmid that expresses the *MFα1* gene under the control of the *GAL1* promoter (*pRS424-P_GAL1_-MFα1*). It also contains the ADGPV integrative plasmid (*pIU-ADGPV*) that expresses the hybrid protein “GPV” under the control of the *ADH1* promoter. The “GPV” construct consists of three domains: the Gal4 DNA binding domain, the human progesterone receptor ligand binding domain and the VP16 activating domain. In the presence of PRO, cells induce expression of αSc.

**Cell#IL3 (ID ALD)** is a cell that produces αSc in the presence of ALD. This cell contains an episomal plasmid that expresses the *MFα1* gene under the control of the *GAL1* promoter (*pRS424-P_GAL1_-MFα1*). It also contains the ADGMV integrative vector (*pIU-ADGMV*) that expresses the hybrid protein “GMV” under the control of the *ADH1* promoter. The “GMV” construct consists of three domains: the Gal4 DNA binding domain, the human mineralocorticoid receptor ligand binding domain and the VP16 activating domain. In the presence of ALD, cells induce expression of αSc.

**Cell#IL4 (ID αCa)** is a cell that produces αSc in the presence of αCa. This cell contains the integrative plasmid (*pRS404-P_FUS1_-MFα1*) that expresses the *MFα1* gene under the control of the *FUS1* promoter. This cell expresses the *C. albicans* pheromone receptor (*CaSTE2*) that has been inserted into the *HIS3* gene locus. In the presence of αCa, this cell triggers *FUS1* promoter transcription to express αSc.

**Cell#IL5 (ID EST)** is a cell that produces αSc in the presence of EST. This cell contains the episomal plasmid *pRS424-P_GAL1_-MFα1* that expresses the *MFα1* gene under the control of the *GAL1* promoter. It also contains the ADGEV integrative vector (*pIU-ADGEV)* that expresses the hybrid protein “GEV” under the control of the *ADH1* promoter. The “GEV” construct consists of three domains: the Gal4 DNA binding domain, the human estradiol receptor ligand binding domain and the VP16 activating domain. In the presence of EST cells induce expression of αSc.

**Cell#IL6 (ID DEX)** is a cell that produces αSc in the presence of DEX. This cell contains the centromeric *pRS416-P_GPD1_-hGR* vector that constitutively expresses the human glucocorticoid receptor. It also contains the centromeric plasmid *pRS413-HEREminp-MFα1*. In the presence of dexamethasone, the glucocorticoid receptor induces transcription of the *MFα1* gene that is under the control of *HERE* sequences. To prevent leakiness, a reduced version of the *GAL1* promoter containing the *Mig2 UAS* was used as a minimal promoter (*minp*). In the presence of DEX cells induce expression of αSc.

**Cell#IL7 (NOT DOX)** is a cell that produces αSc when DOX is absent from the media. IL_2_ cells carry the plasmid *pCM183-MFα1* that expresses *MFα1* under the control of two TetOperators, and in addition, constitutively expresses the Tet Transactivator (tTA).

**Cell#IL8 (NOT PRO)** is a cell that produces αSc in the absence of PRO. The *MFα1* gene is constitutively expressed under the control of the engineered *TEF1i* promoter (*pRS404-P_TEFi_-MFα1*). This cell expresses the LacI repressor from the *GAL1* promoter (*pRS403-P_GAL1_-lacI)* and contains the ADGPV integrative vector (*pIU-ADGPV*) that regulates expression of *lacI* from the *GAL1* promoter in response to progesterone. In the presence of PRO, the LacI repressor is produced and represses the expression αSc.

**Cell#IL9 (NOT ALD)** is a cell that produces αSc in the absence of ALD. The *MFα1* gene is constitutively expressed under the control of the engineered *TEF1i* promoter (*pRS404-P_TEFi_-MFα1*). This cell expresses the LacI repressor from the *GAL1* promoter (*pRS403-P_GAL1_-lacI)* and contains the ADGMV integrative vector (*pIU-ADGMV*) that regulates expression of *lacI* from the *GAL1* promoter in response to aldosterone. In the presence of ALD, the LacI repressor is produced and represses the expression αSc.

**Cell#IL10 (NOT αCa)** is a cell that produces αSc in the absence of αCa. The *MFα1* gene is constitutively expressed under the control of the engineered *TEF1i* promoter (*pRS404-P_TEFi_-MFα1*). This cell expresses the LacI repressor from the *FUS1* promoter (*pRS406-P_FUS1_-lacI)* and contains the *C. albicans* pheromone receptor (*CaSTE2*) inserted into the *HIS3* gene locus. In the presence of αCa, this cell triggers *FUS1* promoter transcription that expresses the LacI repressor and represses the expression of αSc.

**Cell#IL11 (NOT EST)** is a cell that produces αSc in the absence of EST. The *MFα1* gene is constitutively expressed under the control of the engineered *TEF1i* promoter (*pRS404-P_TEFi_-MFα1*). This cell expresses the LacI repressor from the *GAL1* promoter (*pRS405-P_GAL1_-lacI)* and contains the ADGEV integrated vector to regulate the expression of *lacI* from the *GAL1* promoter in response to EST. In the presence of 17-β-estradiol the LacI repressor is produced and represses the expression of αSc.

**Cell#IL12 (NOT DEX)** is a cell that produces αSc in the absence of DEX. The *MFα1* gene is constitutively expressed under the control of the engineered *TEF1i* promoter (*pRS404-P_TEFi_-MFα1*). This cell expresses the LacI repressor under the control of the *HEREminp* construct (*pRS413-HEREminp-lacI*). It also contains the integrative *pRS406-P_GPD1_-hGR* vector. In the presence of DEX, the human glucocorticoid receptor (hGR) induces the transcription of the LacI repressor that blocks expression of αSc.

**Output Layer (OL) cells:** Cells in the Output Layer (OL) are designed to perform a NOT logic: in the absence of the wiring molecule (αSc) they express a fluorescent ssrA tagged reporter protein [11] (yEGFP, OL_1_ or mCherry, OL_2_) or they produce αCa (OL_3_). In the presence of αSc the reporter proteins, or the αCa, are down regulated. In OL_1_ and OL_2_ cells αSc also induces degradation of the reporter proteins using the ClpX/ClpP protease system that targets the ssrA tag [11] (Fig. S2B and C). Cells are mating type MATa and *BAR1* was deleted to increase the sensitivity to pheromone*.*

**Cell#OL1 (NOT GFP)** is a cell that produces yEGFP^ssrA^ in the absence of αSc. The *yEGFP^ssrA^* gene is constitutively expressed under the control of the *TEF1i* promoter (*pRS404-P_TEF1i_-yEGFP^ssrA^)* and the LacI repressor is transcribed from *FUS1* promoter *(pRS405-P_FUS1_-lacI).* In the presence of αSc, the LacI repressor is produced and represses the expression of *yEGFP^ssrA^*. This cell constitutively expresses the protease subunit ClpP under the *ADH1* promoter (*met1::P_ADH1_-ClpP-KanMX*). The ClpX subunit is transcribed from the *FUS1* promoter *(pRS406-P_FUS1_-ClpX)*, which is induced only in the presence of the αSc. When both subunits assemble the protease complex, yEGFP^ssrA^ is degraded*.* This cell also contains the *mCHERRY* gene inserted into the *ENO1* locus (*ENO1::mCHERRY-HphNT*). Expression of mCherry facilitates differentiation of OL_1_ cells (mCherry-positive) from Input Layer cells (mCherry-negative) when mixed (Fig. S5A).

**Cell#OL2 (NOT mCherry)** is a cell that produces mCherry^ssrA^ in the absence of αSc. *mCHERRY^ssrA^* is constitutively expressed under the control of the *TEF1i* promoter (*pRS404/pRS405-P_TEF1i_-mCHERRY^ssrA^;* two copies of mCherry are needed in order to assess fluorescence with a higher degree of confidence). The LacI repressor is transcribed from the *FUS1* promoter *(pRS403-P_FUS1_-lacI).* In the presence of αSc the LacI repressor is produced and represses the expression of *mCHERRY^ssrA^*. This cell constitutively expresses the protease subunit ClpP under the *ADH1* promoter (*met1::P_ADH1_-ClpP-KanMX*). The ClpX subunit is transcribed from the *FUS1* promoter *(pRS406-P_FUS1_-ClpX)*, which is induced only in the presence of αSc. mCherry^ssrA^ is degraded when both subunits assemble the protease complex*.* This cell also expresses the fluorescent protein YFP inserted in the *ENO1* locus (*ENO1::YFP-HphNT*) to distinguish OL_2_ cells (YFP-positive) from the Input Layer cells (YFP-negative) when mixed (Fig. S5C).

**Cell#OL3 (NOT alphaCa)** is a cell that produces αCa in the absence of αSc. *C. albicans* alpha factor is constitutively expressed under the control of the *TEF1i* promoter (*pRS424-P_TEF1i_-CaMFα1*). The LacI repressor is transcribed from the *FUS1* promoter *(pRS405-P_FUS1_-lacI).* In the presence of αSc the LacI repressor is produced and represses the expression of αCa.

**Buffer Layer cell (BL):** Cells in the Buffer Layer (BL) are designed to produce *GFP* in the presence of the αCa. These cells contain *GFP* in the *FUS1* gene locus under its promoter (*fus1::GFP-KanMX),* the *C.* *albicans* pheromone receptor (*CaSTE2*) in the *TRP1* gene locus (*yIP P_TDH3_-Caste2-TRP1)* and *mCHERRY* inserted in the *ENO1* locus (*ENO1::mCHERRY-HphNT*). The design of this cell is based on two fundamental features: 1) sharpen step-like response, and 2) the maximum level of output production is achieved when only a single chamber is activated. Hence, if a given input combination activates more than one chamber this will not be translated into an increase of the output production by the buffer cell. The key aspect of the design of this buffer cell is to obtain a cell that is maximally activated whenever an output is produced by a single chamber.

**Engineered Input Layer cells that respond to hormones:**

We engineered four pairs of IL cells able to respond to four different hormones: 17-β-estradiol (EST), progesterone (PRO), aldosterone (ALD), and dexamethasone (DEX). IL cells able to respond to EST contain the vector *pIU-ADGEV* that expresses a chimeric transcriptional activator (GEV) under the control of the *ADH1* promoter. The chimera construct consists of three domains: the Gal4 DNA binding domain, the human estradiol receptor (hER) hormone binding domain and the VP16 activating domain. The hER belongs to the nuclear hormone family of intracellular receptors. Once activated by the hormone, these receptors translocate into the nucleus and bind to DNA to regulate gene expression. The hormone-binding domain (HBD) of the human estrogen receptor has been previously shown to function as an autonomous regulatory domain in *S. cerevisiae* [12]. When fused to a protein of interest it can subject the activity of this protein to hormone control. This system has been widely used as a good alternative to regulate transcription driven by GAL promoters. Carrying the DNA binding domain of Gal4, this chimerical protein activates transcription from GAL1-10 promoters when 17-β-estradiol is added to the media, even in the presence of glucose. To build IL cells able to respond to progesterone and aldosterone, we copied the above described architecture and substituted the HBD of the human estrogen receptor with the HBD of the human progesterone (hPR) or mineralocorticoid (hMR) receptors. Both the hPR and the hMR belong to the same nuclear hormone family of intracellular receptors. To build the GPV chimera we used the hPR hormone binding domain standing from aminoacid 655 to 933. For the GMV the hMR hormone binding domain standing from aminoacid 705 to 984 was used. IL cells containing the vectors *pIU-ADGPV,* or *pIU-ADGMV*, that expresses the GPV, or GMV, under the control of the *ADH1* promoter are able to respond to PRO and ALD respectively. In ID cells the *GAL* promoter controls the expression of the *MFα1* gene. Therefore, in the presence of the hormone, cells induce the transcription of alpha factor. Correspondingly, in the NOT cells, the *GAL* promoter controls the expression of the LacI repressor. In these cells, in the presence of the hormone, LacI is produced and represses the expression of alpha factor. In the case of cells responding to DEX, cells contain the full length human glucocorticoid receptor (hGR) that is able to induce the transcription of genes under the control of a specific sequence, the *HERE* sequence. In ID cells, a minimal promoter (*minp*) is fused to the *HERE* sequence and controls the expression of the *MFα1* gene. In NOT cells, the same promoter controls the expression of the LacI expression.

**References**

1. Kwok R. Five hard truths for synthetic biology. Nature. 2010; 463: 288-290.
2. Siuti P, Yazbek J, Lu TK. Synthetic circuits integrating logic and memory in living cells. Nat Biotechnol. 2013; 31: 448-452.
3. Bonnet J, Yin P, Ortiz ME, Subsoontorn P, Endy D. Amplifying genetic logic gates. Science. 2013; 340: 599-603.
4. Enderton, Herbert and Enderton, Herbert B. A mathematical introduction to logic. Access Online via Elsevier. 2001.
5. Bender, Edward A. and Williamson, S Gill. A short course in discrete mathematics. DoverPublications.com. 2012.
6. Hill, Frederick J. and Peterson, Gerald R. Introduction to switching theory and logical design. John Wiley & Sons, Inc. 1981.
7. Karnaugh M. The map method for synthesis of combinational logic circuits. American Institute of Electrical Engineers, Part I: Communication and Electronics. 1953; 72: 593-599.
8. McCluskey, Edward J. Introduction to the theory of switching circuits. McGraw-Hill New York. 1965.
9. Macia J, Posas F, Sole RV. Distributed computation: the new wave of synthetic biology devices. Trends Biotechnol. 2012; 30: 342-349.
10. Regot S, Macia J, Conde N, Furukawa K, Kjellen J, Peeters T, Hohmann S, de Nadal E, Posas F, Sole R. Distributed biological computation with multicellular engineered networks. Nature. 2011; 469: 207-211.
11. Grilly C, Stricker J, Pang WL, Bennett MR, Hasty J. A synthetic gene network for tuning protein degradation in Saccharomyces cerevisiae. Mol Syst Biol. 2007; 3: 127.
12. Louvion JF, Havaux-Copf B, Picard D. Fusion of GAL4-VP16 to a steroid-binding domain provides a tool for gratuitous induction of galactose-responsive genes in yeast. Gene. 1993; 131: 129-134.
