# Supplementary material for: Implementation of Complex Biological Logic Circuits Using Spatially Distributed Multicellular Consortia
Source: PLoS Comput Biol. 2016 Feb 1;12(2):e1004685. doi: 10.1371/journal.pcbi.1004685 (PMC4734778; doi:10.1371/journal.pcbi.1004685)
Supplement: S1 Table — (DOCX) [file pcbi.1004685.s002.docx]

**Supporting Table S1.** Yeast strains used in this study.

| **name** | **genotype^*^** | **source** |
| --- | --- | --- |
| IL1 | *MATα ste3::HIS3 mfα1::LEU2 mfα2::KanMX YCpTetO_2_MFα1* | [1] |
| IL2 | *MATα ste3::HIS3 mfα1::LEU2 mfα2::KanMX pCM183-MFα1* | [1] |
| IL3 | *MATα ste3::NatNT mfα1::LEU2 mfα2::KanMX pRS424-P_GAL1_-MFα1 pIU-ADGPV^*^* | this study |
| IL4 | *MATα ste3::NatNT mfα1::LEU2 mfα2::KanMX pRS404-P_TEF1i_-MFα1 pRS403-P_GAL1_-lacI*  *pIU-ADGPV^*^* | this study |
| IL5 | *MATα ste3::NatNT mfα1::LEU2 mfα2::KanMX pRS424-P_GAL1_-MFα1 pIU-ADGMV^**^* | this study |
| IL6 | *MATα ste3::NatNT mfα1::LEU2 mfα2::KanMX pRS404-P_TEF1i_-MFα1 pRS403-P_GAL1_-lacI*  *pIU-ADGMV^**^* | this study |
| IL7 | *MATα ste3::HIS3 mfα1::LEU2 mfα2::KanMX pRS404-P_FUS1_-MFα1 his3::P_GPD1_-CaSTE2-HphNT* | this study |
| IL8 | *MATα ste3::HIS3 mfα1::LEU2 mfα2::KanMX pRS404-P_TEF1i_-MFα1 pRS406-P_FUS1_-lacI his3::P_GPD1_-CaSTE2-HphNT* | this study |
| IL9 | *MATα ste3::NatNT mfα1::LEU2 mfα2::KanMX pRS413-HEREminp-MFα1 pRS416-P_GPD1_-hGR* | this study |
| IL10 | *MATα ste3::NatNT mfα1::LEU2 mfα2::KanMX pRS404-P_TEF1i_-MFα1 pRS413-HEREminp-lacI pRS406-P_GPD1_-hGR* | this study |
| IL11 | *MATα ste3::NatNT mfα1::LEU mfα2::KanMX pRS424-P_GAL1_-MFα1 pIU-ADGEV* | this study |
| IL12 | *MATα ste3::HIS3 mfα1::LEU2 mfα2::KanMX pRS404-P_TEF1i_-MFα1 pRS406-P_GAL1_-lacI*  *pIU-ADGEV* | this study |
| OL1 | *MATa bar1::NatNT met1::P_ADH1_-clpP-KanMX pRS406-P_FUS1_-clpX pRS405-P_FUS1_-lacI*  *pRS404-P_TEF1i_-yEGFP^ssrA^ ENO1-mCHERRY-HphNT* | this study |
| OL2 | *MATa bar1::NatNT met1::P_ADH1_-clpP-KanMX pRS406-P_FUS1_-clpX pRS403-P_FUS1_-lacI*  *pRS404-P_TEF1i_-mCHERRY^ssrA^ pRS405-P_TEF1i_-mCHERRY^ssrA^ ENO1-YFP-HphNT* | this study |
| OL3 | *MATa bar1::NatNT pRS405-P_FUS1_-lacI pRS424-P_TEF1i_-CaMFα1* | this study |
| BL | *MATa bar1::HIS3 ste2::URA3 yIP P_TDH3_-Caste2-TRP fus1::GFP-KanMX*  *ENO1-mCHERRY-HphNT* | this study |
| ***** Strain background W303 (*ade2-1 his3-11,15 leu2-3,112 trp1-1 ura3-1 can1-100*) | | |

**References**

1. Regot S, Macia J, Conde N, Furukawa K, Kjellen J, Peeters T, Hohmann S, de Nadal E, Posas F, Sole R. Distributed biological computation with multicellular engineered networks. Nature. 2011; 469: 207-211.
