# Supplementary material for: Implementation of Complex Biological Logic Circuits Using Spatially Distributed Multicellular Consortia
Source: PLoS Comput Biol. 2016 Feb 1;12(2):e1004685. doi: 10.1371/journal.pcbi.1004685 (PMC4734778; doi:10.1371/journal.pcbi.1004685)
Supplement: S2 Table — (DOCX) [file pcbi.1004685.s003.docx]

**Supporting Table S2.** Plasmids used in this study.

| **name** | **description** | **source** |
| --- | --- | --- |
| *pRS406-P_FUS1_-clpX* | Integrating *URA3 p _FUS1_-clpX* | this study and [1] |
| *pRS405-P_FUS1_-lacI* | Integrating *LEU2 P_FUS1_-lacI* | this study |
| *pRS403-P_FUS1_-lacI* | Integrating *HIS3 P_FUS1_-lacI* | this study |
| *pYM-N6-P_ADH1_-clpP-KanMX* | Integrating *MET1 P_ADH1_-clpP* Geneticin resistance | this study and [1,2] |
| *pRS404-P_TEF1i_-yEGFP^ssrA^* | Integrating *TRP1 P_TEF1_-OplacI-yEGFP^ssrA^* | this study and [1] |
| *pRS404-P_TEF1i_-mCHERRY^ssrA^* | Integrating *TRP1 P_TEF1_-OplacI-mCHERRY^ssrA^* | this study |
| *pRS405-P_TEF1i_-mCHERRY^ssrA^* | Integrating *LEU2 P_TEF1_-OplacI-mCHERRY^ssrA^* | this study |
| *pBS35-mCHERRY-HphNT* | Integrating *ENO1* terminator *mCHERRY* HygromycinB resistance | this study and [2] |
| *pYM40-YFP-HphNT* | Integrating *ENO1* terminator *YFP* HygromycinB resistance | this study and [2] |
| *YCpTetO_2_MFα1* | CEN *TRP1* reverse tTA transactivator *TetO_2_MFα1* | [3] |
| *pRS424-P_GAL1_-MFα1* | 2 micron *TRP1 P_GAL1_-MFα1* | this study |
| *pRS404-P_FUS1_-MFα1* | Integrating *TRP1 P_FUS1_-MFα1* | this study |
| *pRS413-HEREminp-MFα1* | CEN *HIS3 HEREminp-MFα1* | this study and [4] |
| *pCM183-MFα1* | CEN *TRP1* tTA transactivator *TetO_2_MFα1* | [3] |
| *pRS404-P_TEF1i_-MFα1* | Integrating *TRP1 P_TEF1_-OplacI-MFα1* | this study |
| *pRS403-P_GAL1_-lacI* | Integrating *HIS3 P_GAL1_-lacI* | this study |
| *pRS406-P_GAL1_-lacI* | Integrating *URA3 P_GAL1_-lacI* | this study |
| *pRS406-P_FUS1_-lacI* | Integrating *URA3 P_FUS1_-lacI* | this study |
| *pRS413-HEREminp-lacI* | CEN *HIS3 HEREminp-lacI* | this study and [4] |
| *pFA6a-hphNT1-P_GPD1_-CaSTE2* | Integrating *HIS3 P_GPD1_-CaSTE2* HygromycinB resistance | this study and [2,5] |
| *pIU-ADGPV* | Integrating *URA3 P_ADH1_-[GAL4DBD-hPR LBD-VP16]*^*^ | this study |
| *pIU-ADGMV* | Integrating *URA3 P_ADH1_-[GAL4DBD-hMR LBD-VP16]* ^†^ | this study |
| *pIU-ADGEV* | Integrating *URA3 P_ADH1_-[GAL4DBD-hER LBD-VP16]* | [6] |
| *pRS416-P_GPD1_-hGR* | CEN *URA3 P_GPD1_-hGR* | this study and [4] |
| *pRS406-P_GPD1_-hGR* | Integrating *URA3 P_GPD1_-hGR* | this study and [4] |
| *yIP P_TDH3_-Caste2-TRP* | Integrating *TRP1 P_TDH3_-CaSTE2* | this study |
| *fus1::GFP-KanMX* | Integrating *FUS1p locus GFP* Geneticin resistance | this study |
| *pRS424-P_TEF1i_-CaMFα1* | 2 micron *TRP1 P_TEF1_-OplacI-CandidaAlbicansMFα1* | this study |
| ^*^The hPR ligand binding domain stands from aminoacid 655 to 933. | | |
| ^†^The hMR ligand binding domain stands from aminoacid 705 to 984. | | |

**References**

1. Grilly C, Stricker J, Pang WL, Bennett MR, Hasty J. A synthetic gene network for tuning protein degradation in Saccharomyces cerevisiae. Mol Syst Biol. 2007; 3: 127.
2. Janke C, Magiera MM, Rathfelder N, Taxis C, Reber S, Maekawa H, Moreno-Borchart A, Doenges G, Schwob E, Schiebel E, Knop M. A versatile toolbox for PCR-based tagging of yeast genes: new fluorescent proteins, more markers and promoter substitution cassettes. Yeast. 2004; 21: 947-962.
3. Regot S, Macia J, Conde N, Furukawa K, Kjellen J, Peeters T, Hohmann S, de Nadal E, Posas F, Sole R. Distributed biological computation with multicellular engineered networks. Nature. 2011; 469: 207-211.
4. Miller CA, III, Tan X, Wilson M, Bhattacharyya S, Ludwig S. Single plasmids expressing human steroid hormone receptors and a reporter gene for use in yeast signaling assays. Plasmid. 2010; 63: 73-78.
5. Janiak AM, Sargsyan H, Russo J, Naider F, Hauser M, Becker JM Functional expression of the Candida albicans alpha-factor receptor in Saccharomyces cerevisiae. Fungal Genet Biol. 2005; 42: 328-338.
6. Louvion JF, Havaux-Copf B, Picard D. Fusion of GAL4-VP16 to a steroid-binding domain provides a tool for gratuitous induction of galactose-responsive genes in yeast. Gene. 1993; 131: 129-134.
