# Supplementary material for: Implementation of Complex Biological Logic Circuits Using Spatially Distributed Multicellular Consortia
Source: PLoS Comput Biol. 2016 Feb 1;12(2):e1004685. doi: 10.1371/journal.pcbi.1004685 (PMC4734778; doi:10.1371/journal.pcbi.1004685)
Supplement: S3 Table — (DOCX) [file pcbi.1004685.s004.docx]

**Supporting Table S3.** Fitting parameters and correlation coefficient r.

| **Cell** | **γ** | **α** | **ω** | **n** | ***r*** |
| --- | --- | --- | --- | --- | --- |
| IL1 | 65.3 | 0.26 | 5.66·10^-11^ | 3.7 | 0.994 |
| IL2 | 10 | 8.7 | 2.5·10^-3^ | 1.7 | 0.998 |
| IL3 | 97 | 0 | 8.2·10^-5^ | 3.5 | 0.999 |
| IL4 | 4.1 | 21.7 | 1.5·10^-2^ | 1.5 | 0.997 |
| IL5 | 93.5 | 0.0107 | 8.01·10^-11^ | 2.9 | 0.999 |
| IL6 | 20.2 | 4.4 | 3.5·10^-7^ | 1,98 | 0.993 |
| IL7 | 86.5 | 0.208 | 1.52·10^-6^ | 1.4 | 0.994 |
| IL8 | 9 | 9.4 | 4·10^-10^ | 2.9 | 0.996 |
| IL9 | 89.7 | 0.0245 | 2.89·10^-9^ | 7.9 | 0.995 |
| IL10 | 5.9 | 14.1 | 3.6·10^-6^ | 3.1 | 0.994 |
| IL11 | 95 | 0 | 1.62·10^-5^ | 3.1 | 0.998 |
| IL12 | 2.1 | 36,6 | 1·10^-3^ | 1.45 | 0.996 |
| OL1_%GFP_ | 98.01 | 0.0102 | 1.5·10^-14^ | 4.3 | 0.998 |
| OL1_GFPa.u._ | 95 | 0.0105 | 4.8·10^-14^ | 4.3 | 0.996 |
| OL2 | 95 | 0.105 | 3.39·10^-11^ | 2.65 | 0.999 |
| OL3 | 85 | 0.1335 | 1·10^-5^ | 1.6 | 0.998 |
| BL | 4 | 24.5 | 1.5·10^-38^ | 10 | 0.998 |
