# Supplementary figures and images for: Implementation of Complex Biological Logic Circuits Using Spatially Distributed Multicellular Consortia
Source: PLoS Comput Biol. 2016 Feb 1;12(2):e1004685. doi: 10.1371/journal.pcbi.1004685 (PMC4734778; doi:10.1371/journal.pcbi.1004685)

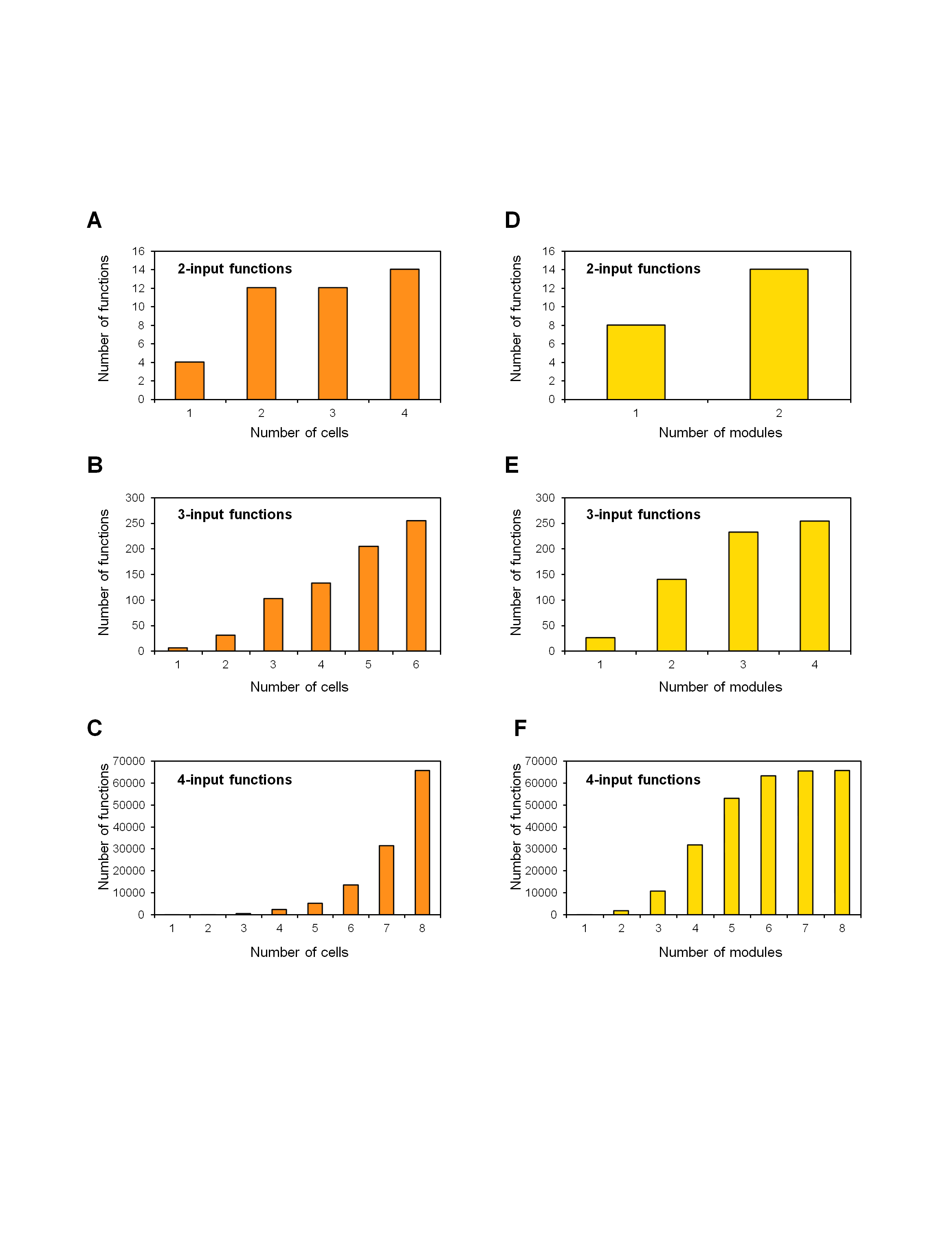

Supplement: S1 Fig — (A, B, C) Dependence of the number of possible implementable functions with respect to the number of different cells required. Data for functions with 2, 3 and 4 inputs are shown. (D, E, F) Dependence of the number of possible implementable functions with respect to the number of different modules (consortia) required. Data for functions with 2, 3 and 4 inputs are shown. (TIF) [file pcbi.1004685.s005.TIF]

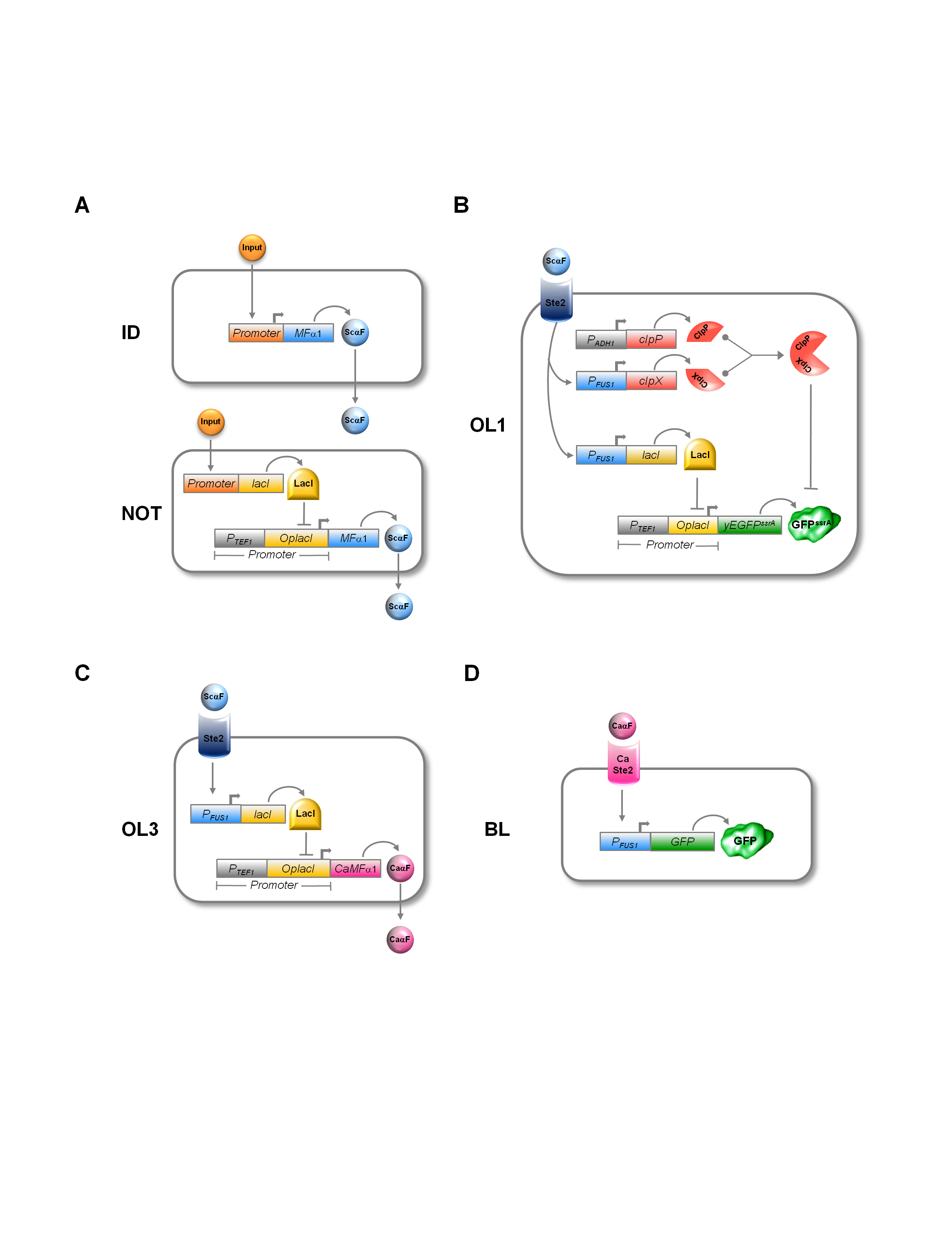

Supplement: S2 Fig — (A) Identity cells (ID; top) express the S. cerevisiae alpha factor pheromone under the control of an input-inducible promoter. NOT cells (NOT; bottom) constitutively express this pheromone under the control of a modified TEF1 promoter (TEF1-OplacI). In the presence of input, S. cerevisiae alpha factor expression is repressed by LacI. (B) An ssrA-tagged version of yEGFP was expressed under the control of the TEF1i promoter. The ssrA tag allows the Clp protease complex to recognize yEGFPsrrA and induce its degradation. The protease subunit ClpP is constitutively transcribed under the ADH1 promoter, whereas the ClpX subunit and the LacI repressor were expressed under the control of the FUS1 promoter, which is induced by S. cerevisiae alpha factor. (C) OL3 cells constitutively express the C. albicans alpha pheromone under the control of a modified TEF1 promoter (TEF1-OplacI). The LacI repressor was expressed under the control of the FUS1 promoter, which is induced by S. cerevisiae alpha factor. (D) Buffer Layer (BL) cells express the C. albicans pheromone receptor STE2 and produce GFP in the presence of C. albicans alpha factor. (TIF) [file pcbi.1004685.s006.TIF]

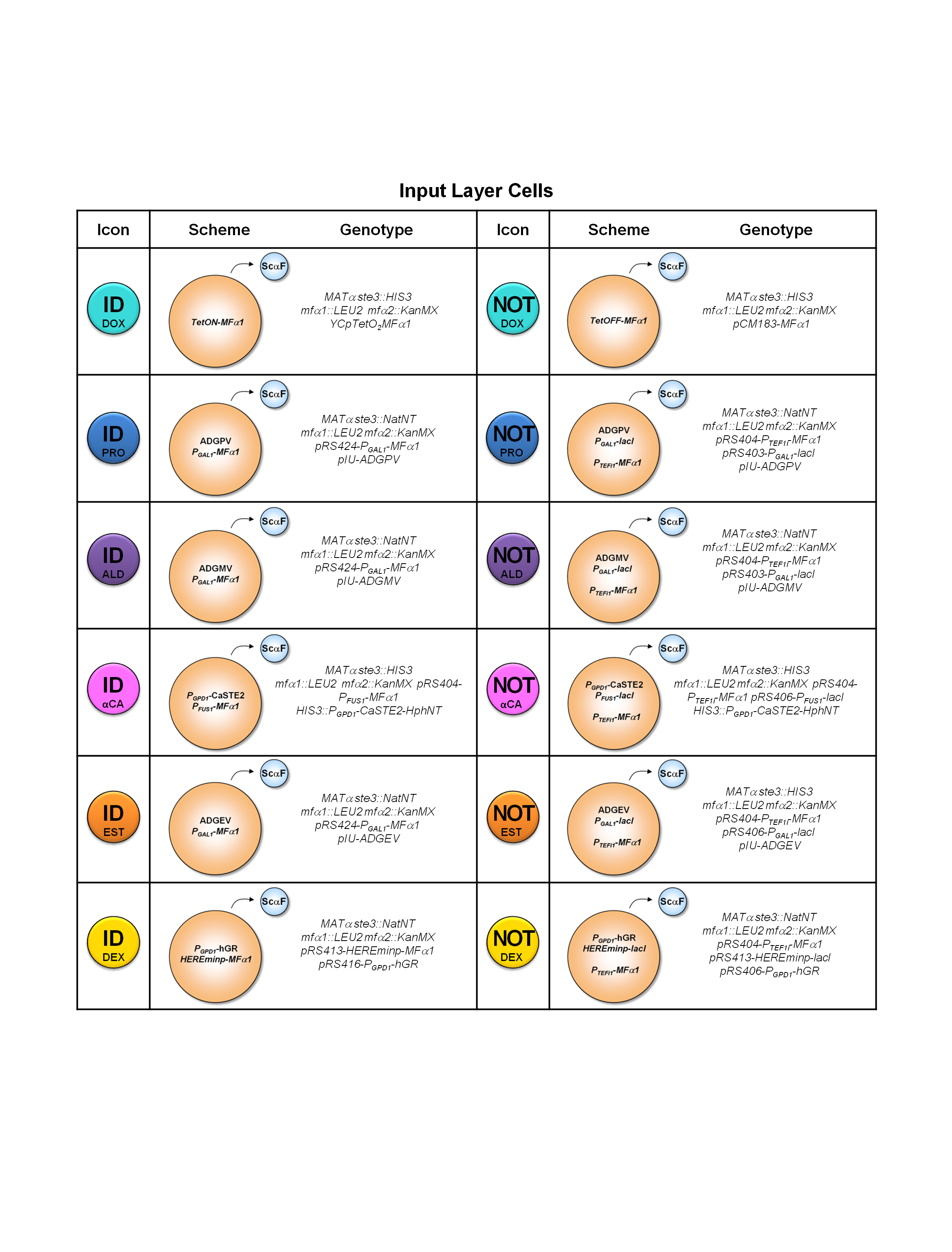

Supplement: S3 Fig — Cells in the library respond to six different inputs (DOX; doxycycline, PRO; progesterone, ALD; aldosterone, αCa; C. albicans alpha factor, EST; 17-β-estradiol, DEX; dexamethasone) with two different logics. In the presence of the input, Identity cells (ID, left) express S. cerevisiae alpha factor, whereas NOT cells (NOT, right) repress pheromone production in response to stimuli. All cells are W303 derivatives. (TIF) [file pcbi.1004685.s007.TIF]

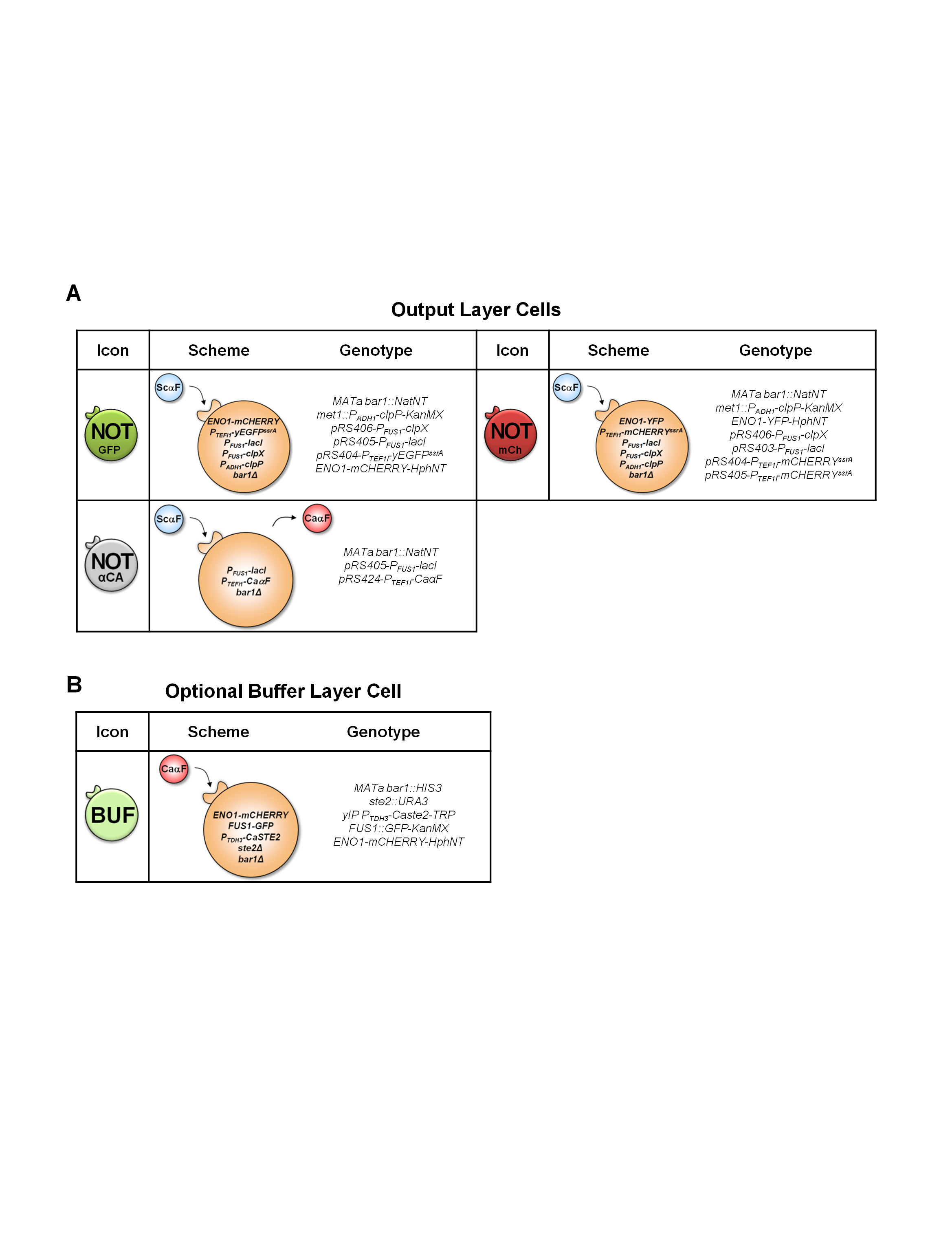

Supplement: S4 Fig — (A) The Output Layer cells sense S. cerevisiae alpha factor and shut down the expression of a fluorescent protein (GFP, mCherry) or the production of C. albicans alpha factor. All cells are W303 derivatives. (B) The Buffer Layer cell sense C. albicans alpha factor and produce GFP. Cell is W303 derivative. (TIF) [file pcbi.1004685.s008.TIF]

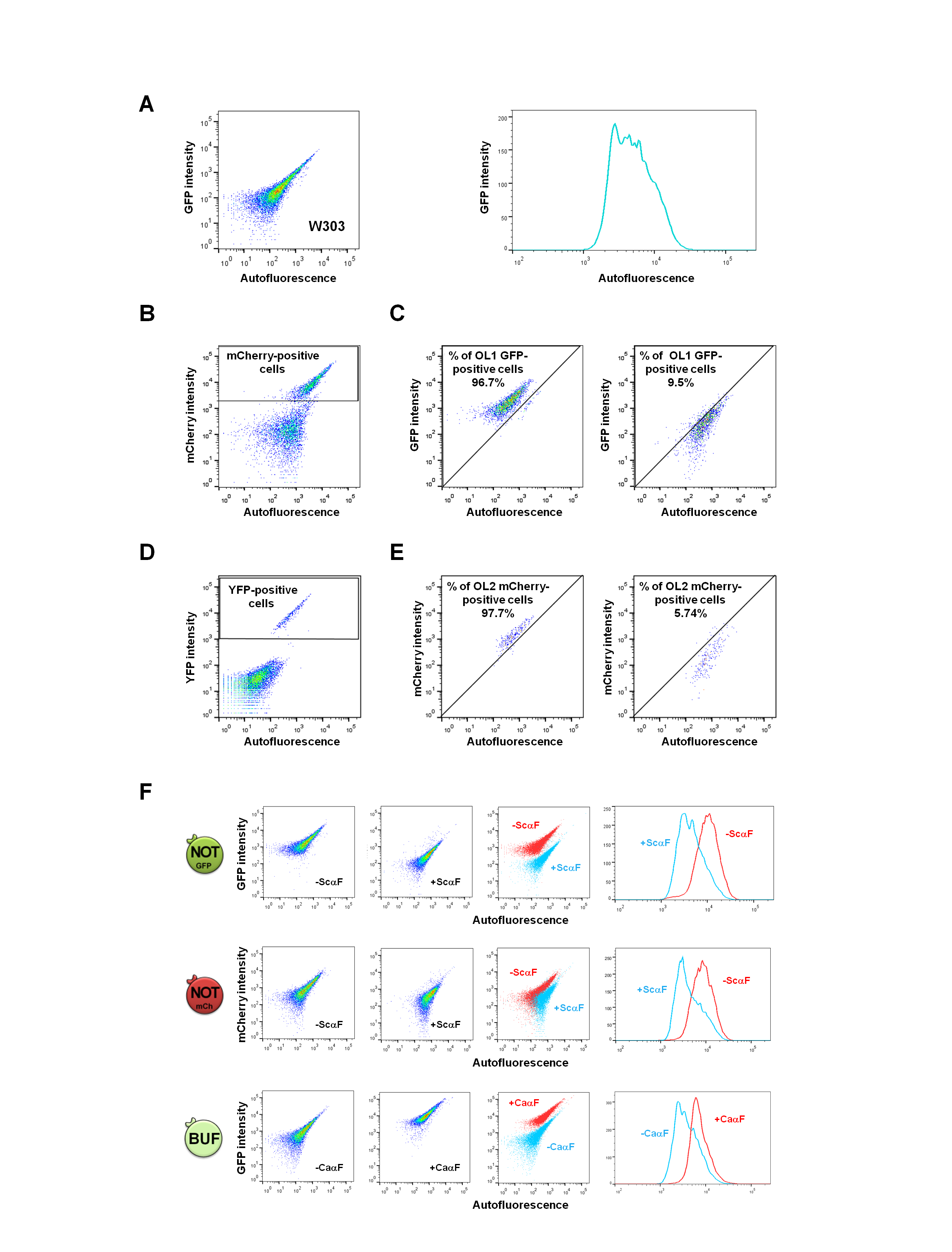

Supplement: S5 Fig — Fluorescence from Output Layer and Buffer Layer cells was assessed by flow cytometry. A total of 10.000 cells were analyzed. (A) Representative FACS plot of a wild type W303 cells. (B) Panel shows mCherry intensity (Y axis) versus autofluorescence (X axis) and allows selecting the OL1, or BL, cells (mCherry positive) from the Input Layer cells (mCherry negative). (C) Selected OL1, or BL, mCherry cells were analyzed by their GFP expression (Y axis) versus autofluorescence (X axis). Two examples are given: a GFP positive sample (left) and GFP negative one (right). (D) OL2 cells are analyzed as in A, using the YFP channel to select them from the Input Layer cells. (E) Selected YFP cells were assessed by their mCherry expression. Two examples are given: a mCherry positive sample (left) and mCherry negative one (right). (F) Population density and histograms plots of fluorescence intensities of OL1, OL2 and BUF cells. Histograms plots are compared to density plots in presence or absence of the corresponding alpha factor. (TIF) [file pcbi.1004685.s009.TIF]

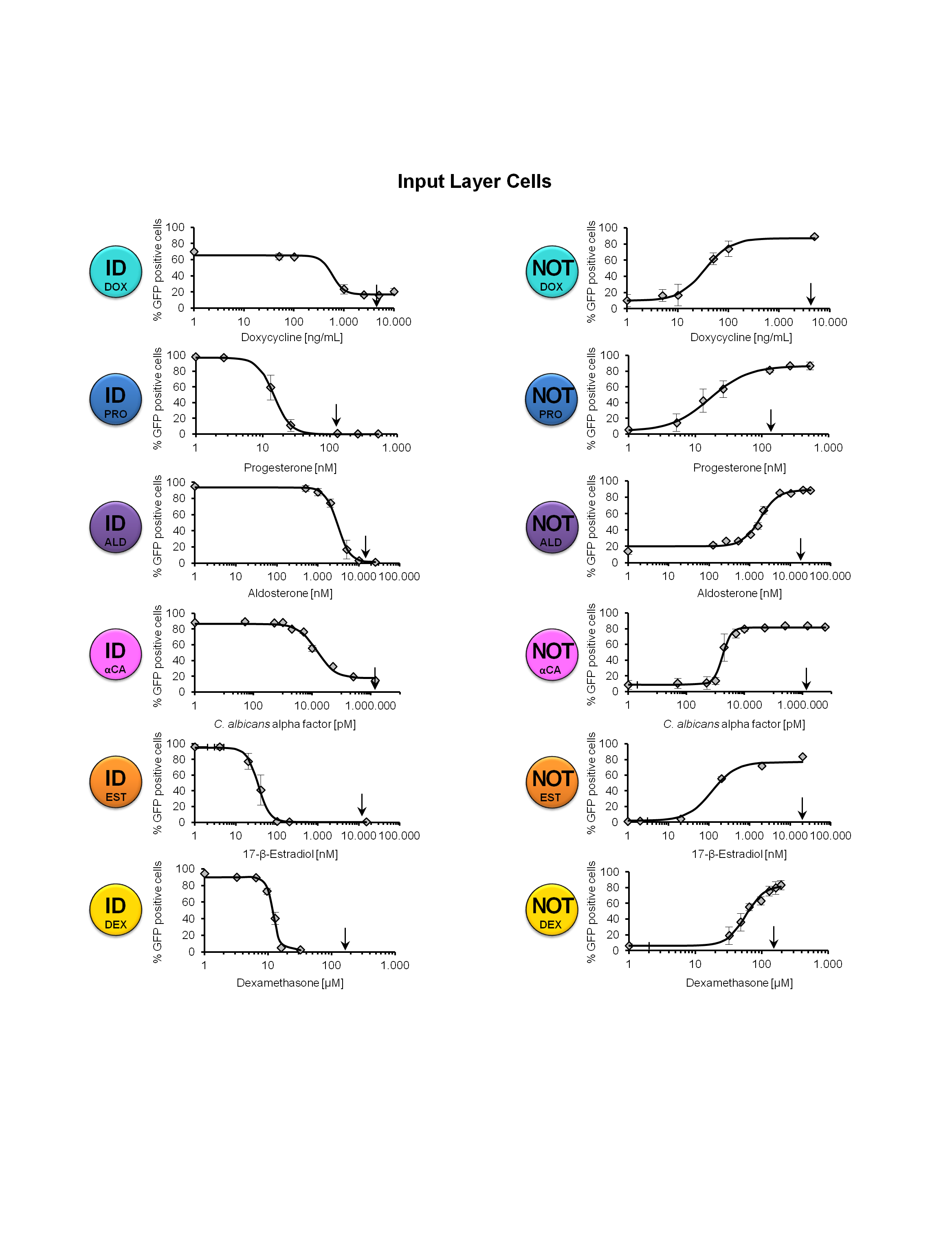

Supplement: S6 Fig — Input Layer cells were mixed with the Output Layer GFP cells (OL1) and treated with different inputs concentrations. Samples were incubated for 4h at 30°C and analyzed by FACS. Data are expressed as the percentage of GFP positive cells and represent the mean and standard deviation of three independent experiments. Arrows indicate the working concentrations of inputs. (TIF) [file pcbi.1004685.s010.TIF]

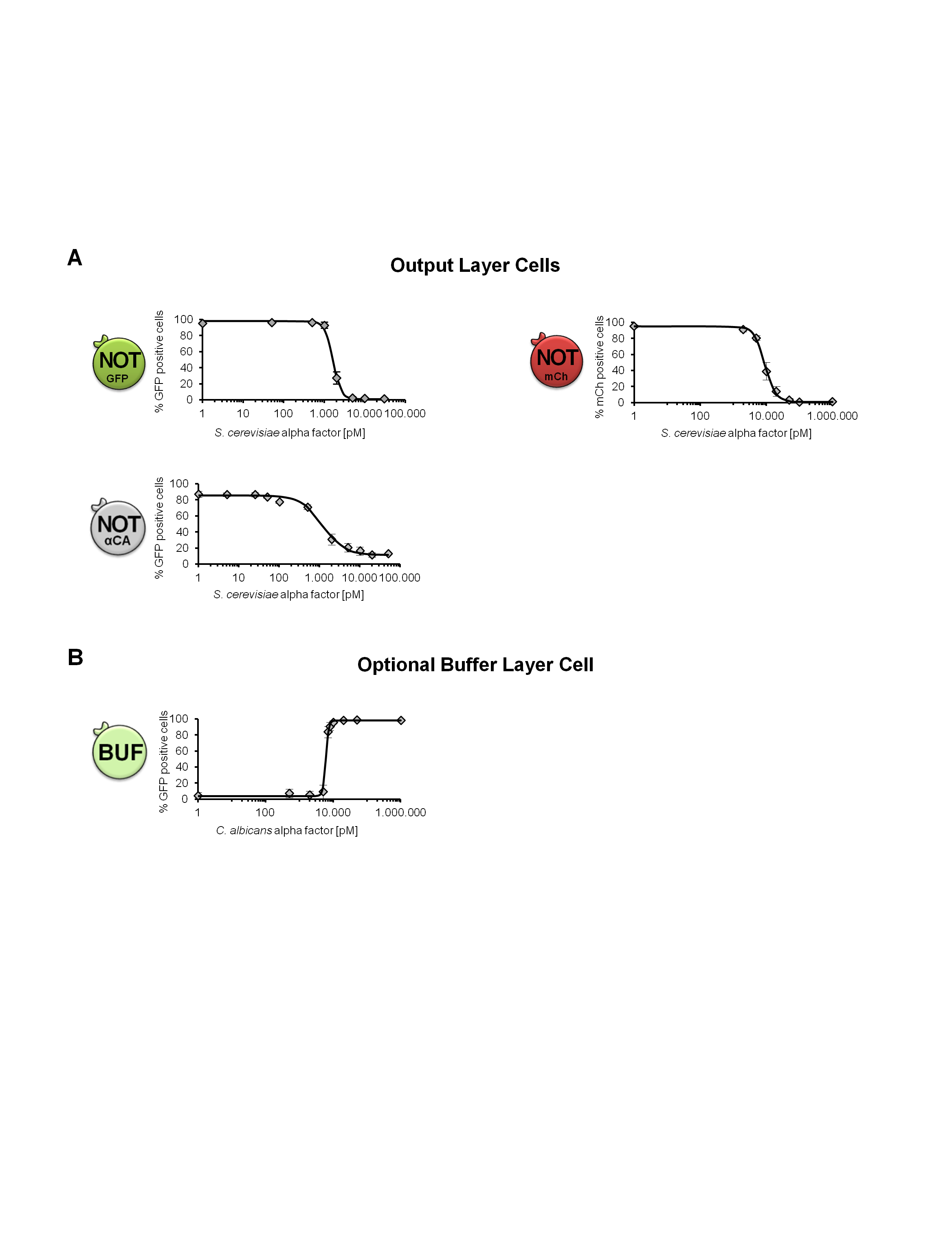

Supplement: S7 Fig — (A) Output Layer cells OL1 (upper left) and OL2 (upper right) were incubated with different concentrations of S. cerevisiae alpha-factor and analyzed as in S6 Fig. Output Layer cells OL3 (lower left) were incubated with Buffer Layer cells in the presence of different concentrations of S. cerevisiae alpha-factor and analyzed as in S6 Fig. (B) Buffer Layer cells were incubated with different concentrations of C. albicans alpha factor and analyzed as in S6 Fig. (TIF) [file pcbi.1004685.s011.TIF]

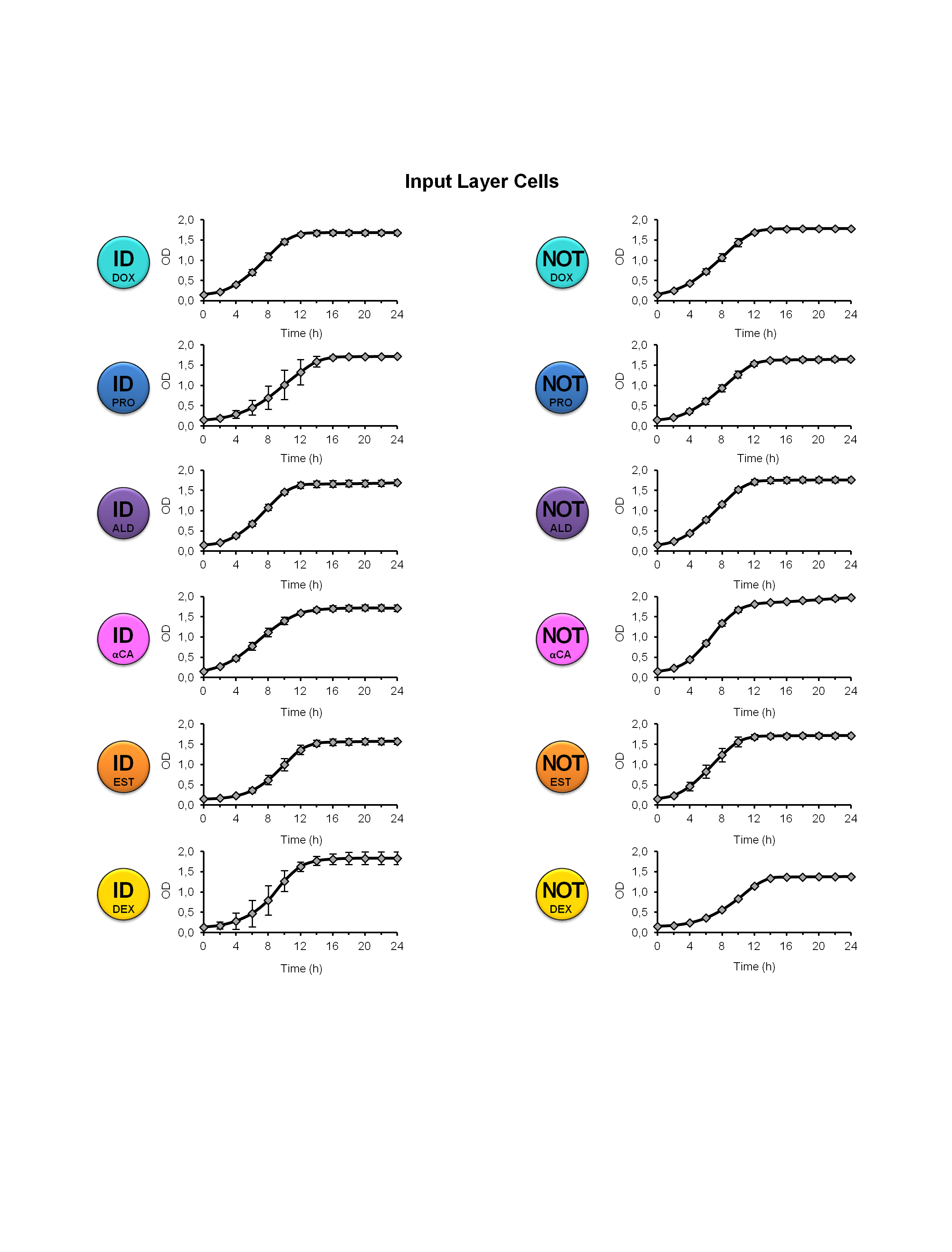

Supplement: S8 Fig — Exponential cultures of Input Layer cells were diluted to OD660 nm ≈ 0.02 and their growth curve was measured using Synergy H1 BioTeK for 24 h. Data represent the mean and standard deviation of three independent experiments. (TIF) [file pcbi.1004685.s012.TIF]

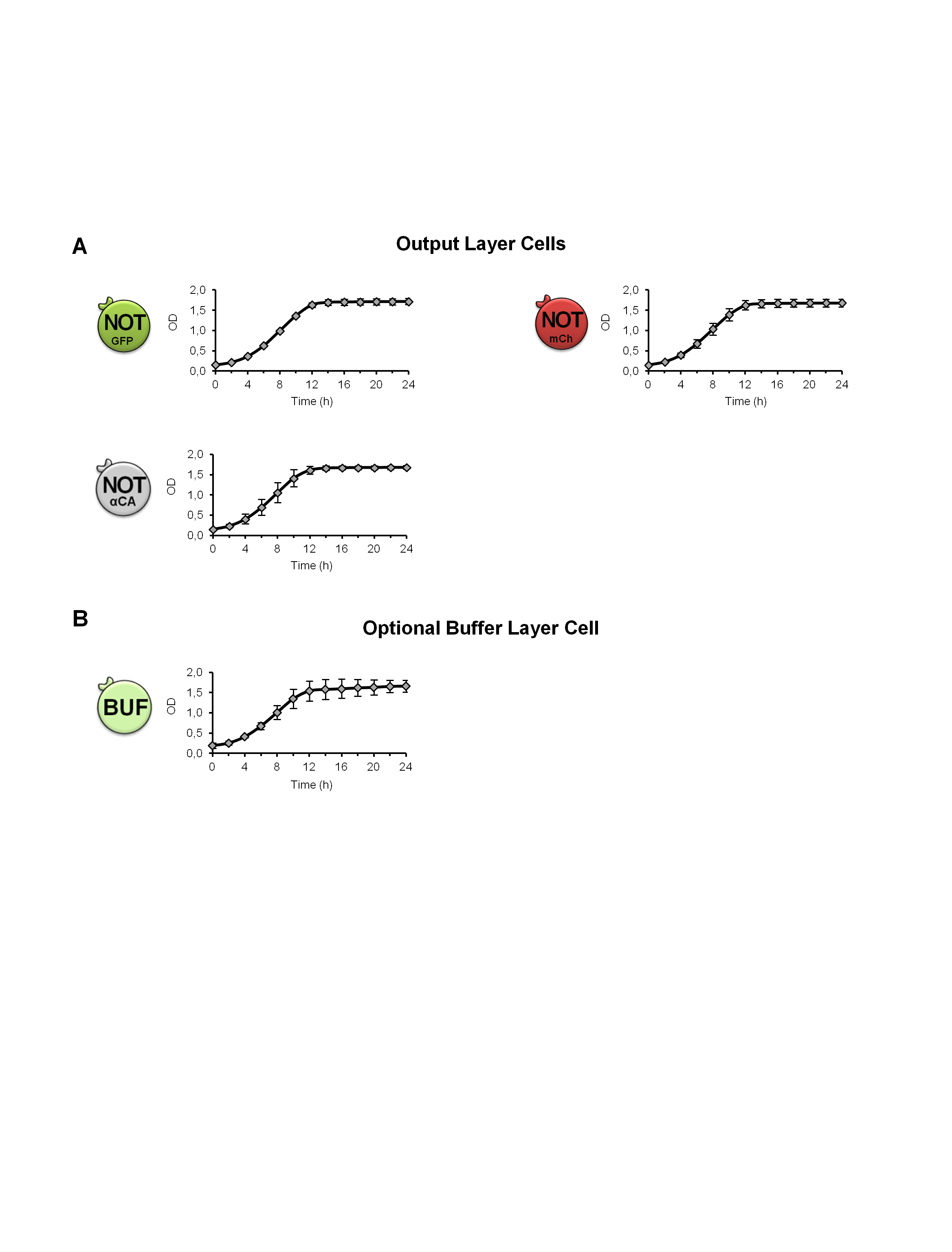

Supplement: S9 Fig — (A) Output Layer cells OL1 (upper left), OL2 (upper right) and OL3 (lower left) growth curve was measured as in S8 Fig. (B) Buffer Layer cells growth curve was measured as in S8 Fig. (TIF) [file pcbi.1004685.s013.TIF]

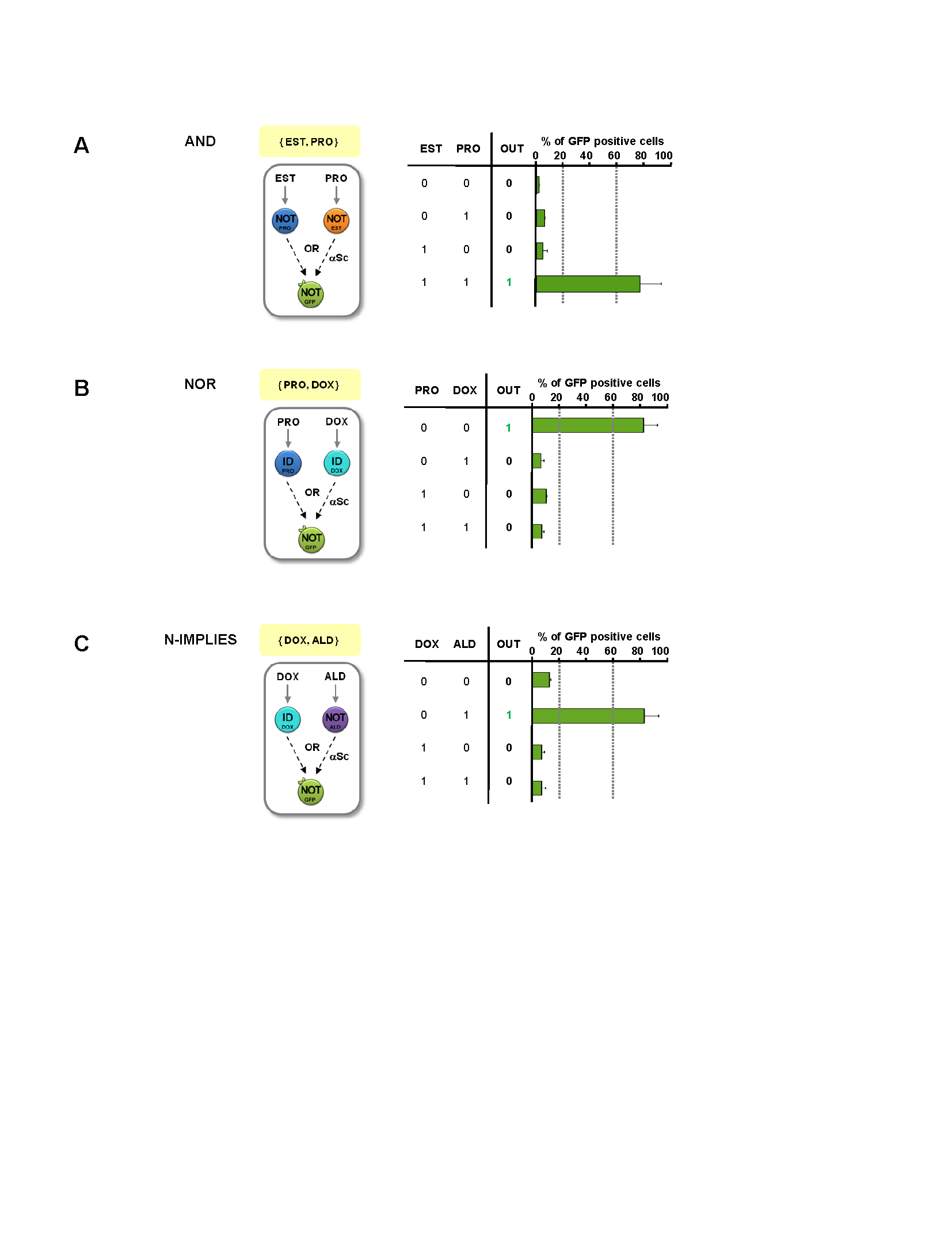

Supplement: S10 Fig — (A) AND gate. (B) NOR gate. C) N-IMPLIES gate. Schematic representation of the cells used in the circuits (left). Truth table (middle). Percentage of OL1 GFP-positive cells (right). Cells were mixed proportionally and treated with different combinations of inputs. After computing, for each combination of inputs, the percentage of OL1 GFP positive cells was analyzed using FACS. Data were analyzed and processed as described in Material and Methods and represent the mean and standard error of three independent experiments. (TIFF) [file pcbi.1004685.s014.tiff]

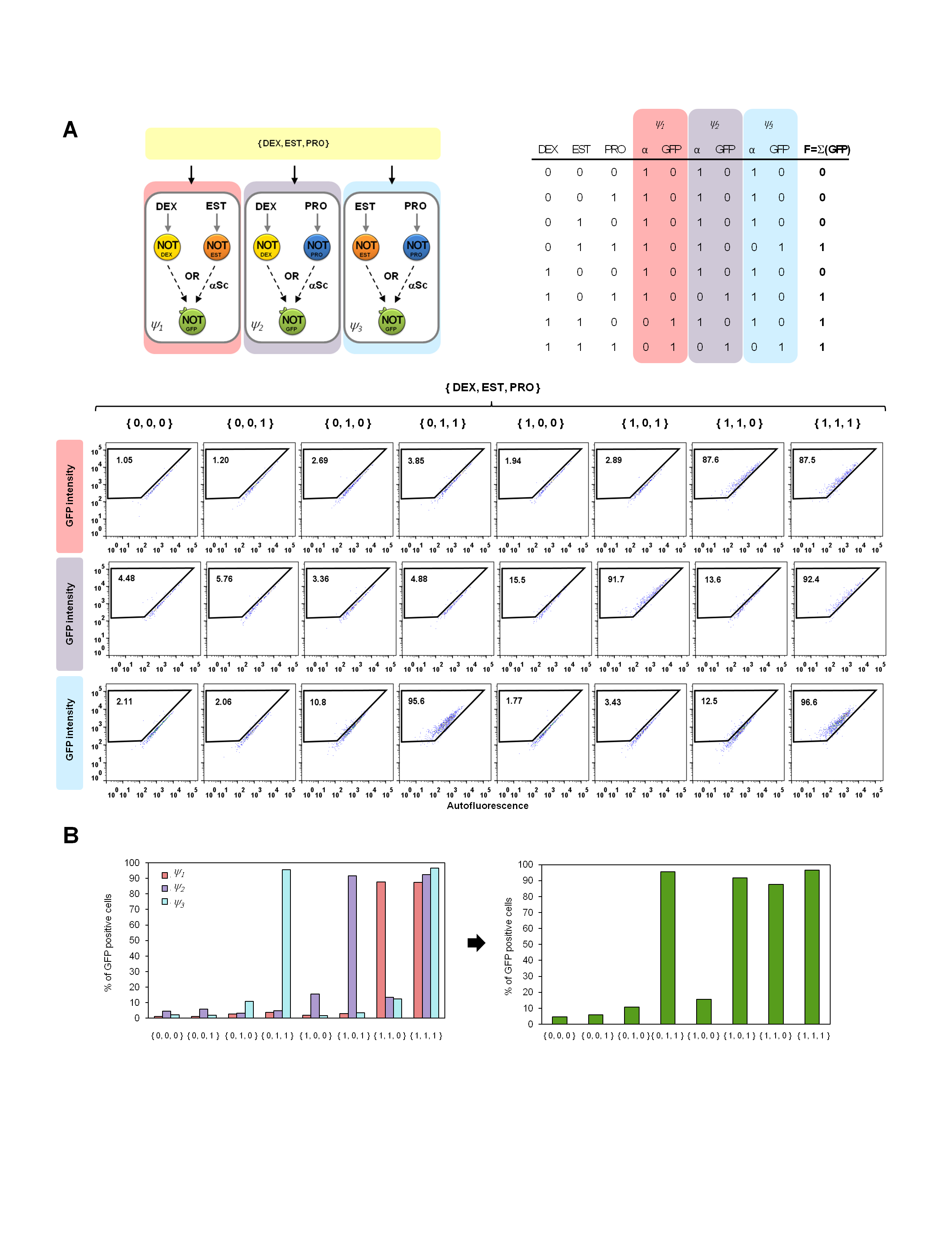

Supplement: S11 Fig — (A) The quantification of one majority rule experiment is showed as an illustrative example of all circuits’ quantification and data treatment. For every chamber, and for each combination of inputs, fluorescence intensity of the subsets of OL cells was measured versus autofluorescence. Data are expressed as percentage of GFP positive cells and analyzed using FlowJo. (B) When more than one consortium gave a positive fluorescent signal we choose the highest value as the final circuit’s output. (TIF) [file pcbi.1004685.s015.TIF]

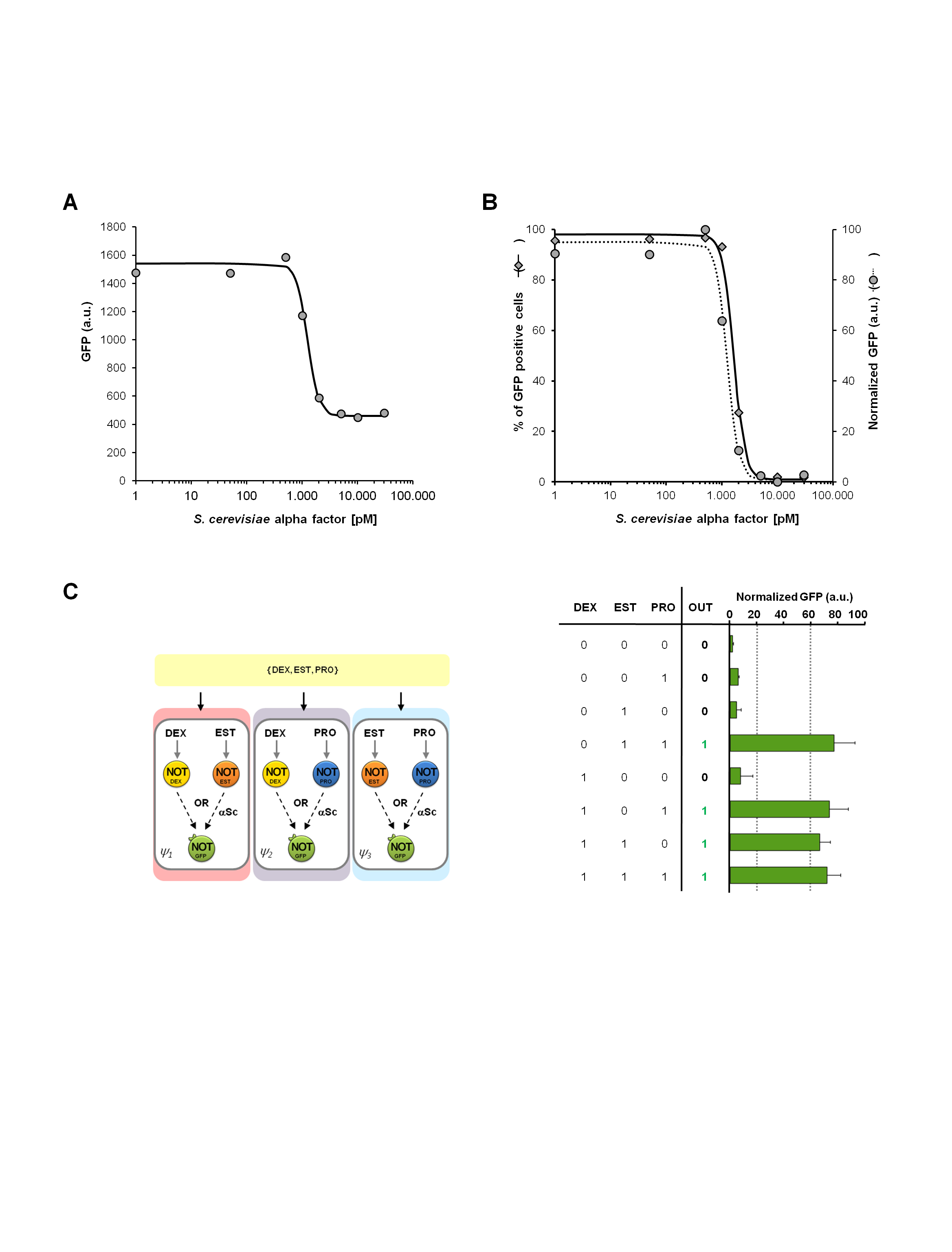

Supplement: S12 Fig — (A) Transfer function data of the OL1 cells expressed as GFP a.u. Values ranges from maximum of 1600 to a minimum of 450 GFP a.u and the curve presents a step like shape. Experimental transfer function data were fitted to a Hill equation as described in S1 Text. (B) Normalized GFP (a.u.) transfer function of OL1 cells (round circles) overlapped with the same transfer function where data are expressed as % of GFP positive cells (diamonds). Experimental transfer functions data were fitted to a Hill equation as described in S1 Text (GFP a.u., dotted line; % GFP, straight line). Both transfer functions exhibit a proper behavior that allows the definition of a clear threshold between 0 and 1 logic states. (C) Schematic representation and spatial distribution of the cells used in the majority rule circuit (left). Truth table (middle). Results of the majority rule circuit presented in Fig 3C, green bars, analyzed as normalized GFP (a.u.) (right). Data represent the mean and standard error of three independent experiments. (TIF) [file pcbi.1004685.s016.TIF]

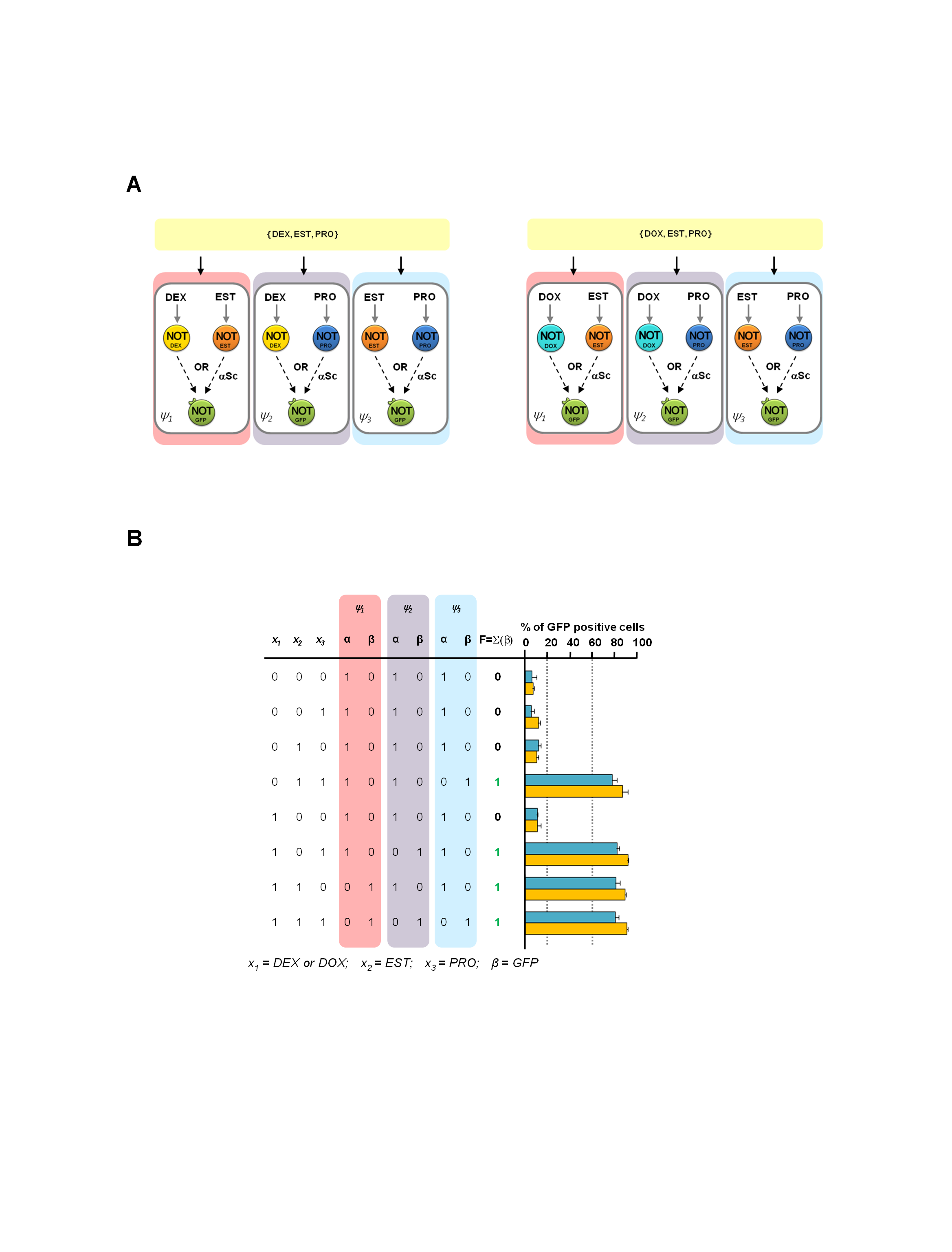

Supplement: S13 Fig — (A) Schematic representation and spatial distribution of the cells used in the majority rule circuit. The circuit is the same as in Fig 3 except here we used IL7 cells, which respond to DOX (right), instead of the previously used IL12 cells, which respond to DEX (left). (B) Truth table (left) and percentage of FACS GFP positive cells (right). Yellow bars refer to the circuit built with IL12 (DEX) visualized also in Fig 3C, green; cyan refers to the same circuit built with IL7 (DOX). Data represent the mean and standard error of three independent experiments. (TIF) [file pcbi.1004685.s017.TIF]

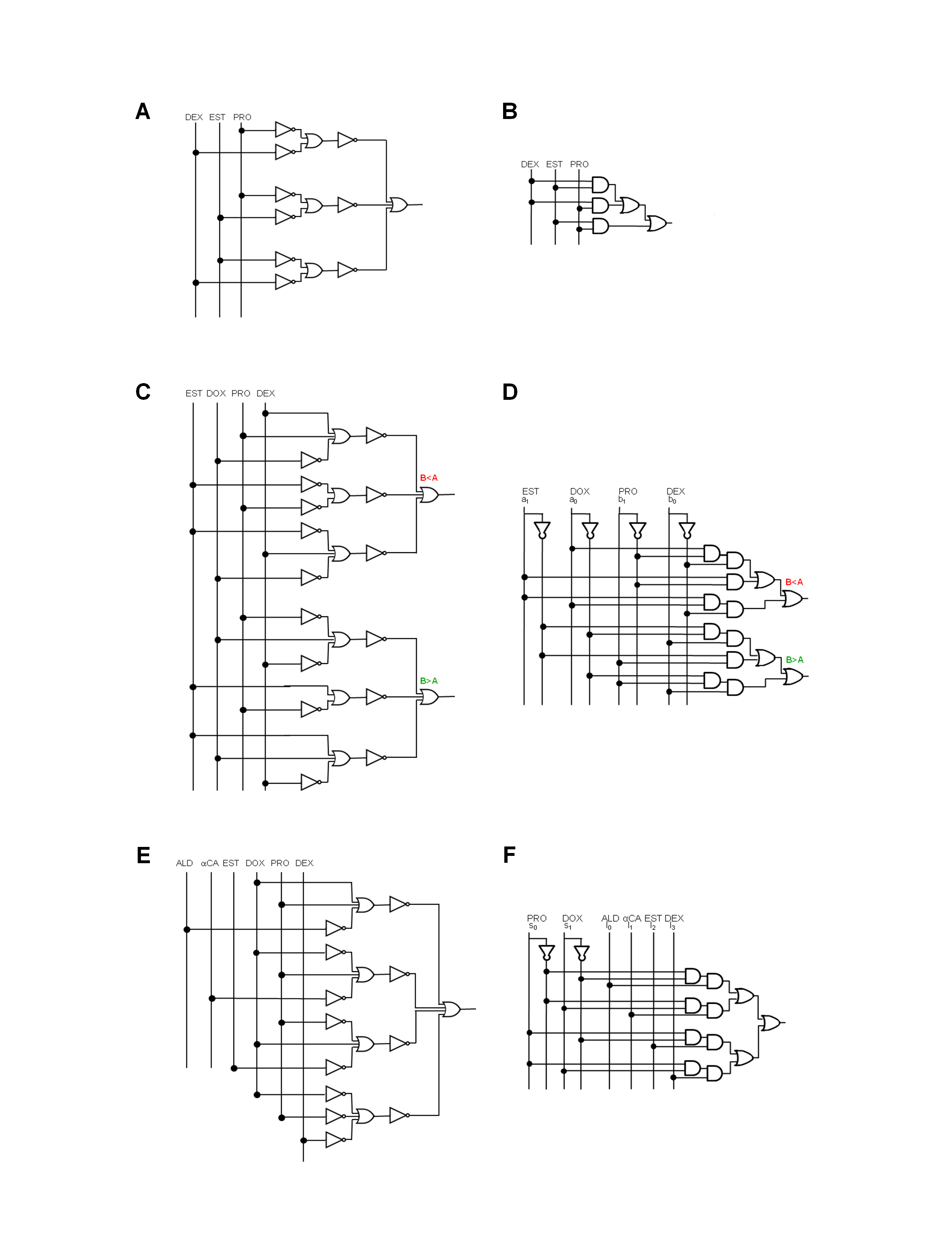

Supplement: S14 Fig — Despite the logic representation of circuits based on ILF involves NOT and OR logic gates, only NOT gates are genetically implemented. OR logic is implicitly implemented by spatial segregation of the consortia. In each consortium, the IL cells produce the same wire molecule in a shared environment thus implementing an implicit OR logic gate. Combining this OR gate with the NOT gate of the OL cells results in a multi-input NOR gate. (A) ILF design of the majority rule circuit. (B) Standard design of the majority rule circuit. (C) ILF design of the 2-bit magnitude comparator. (D) Standard design of the 2-bit magnitude comparator. (E) ILF design of the multiplexer MUX4to1. (F) Standard design of the multiplexer MUX4to1. (TIF) [file pcbi.1004685.s018.TIF]

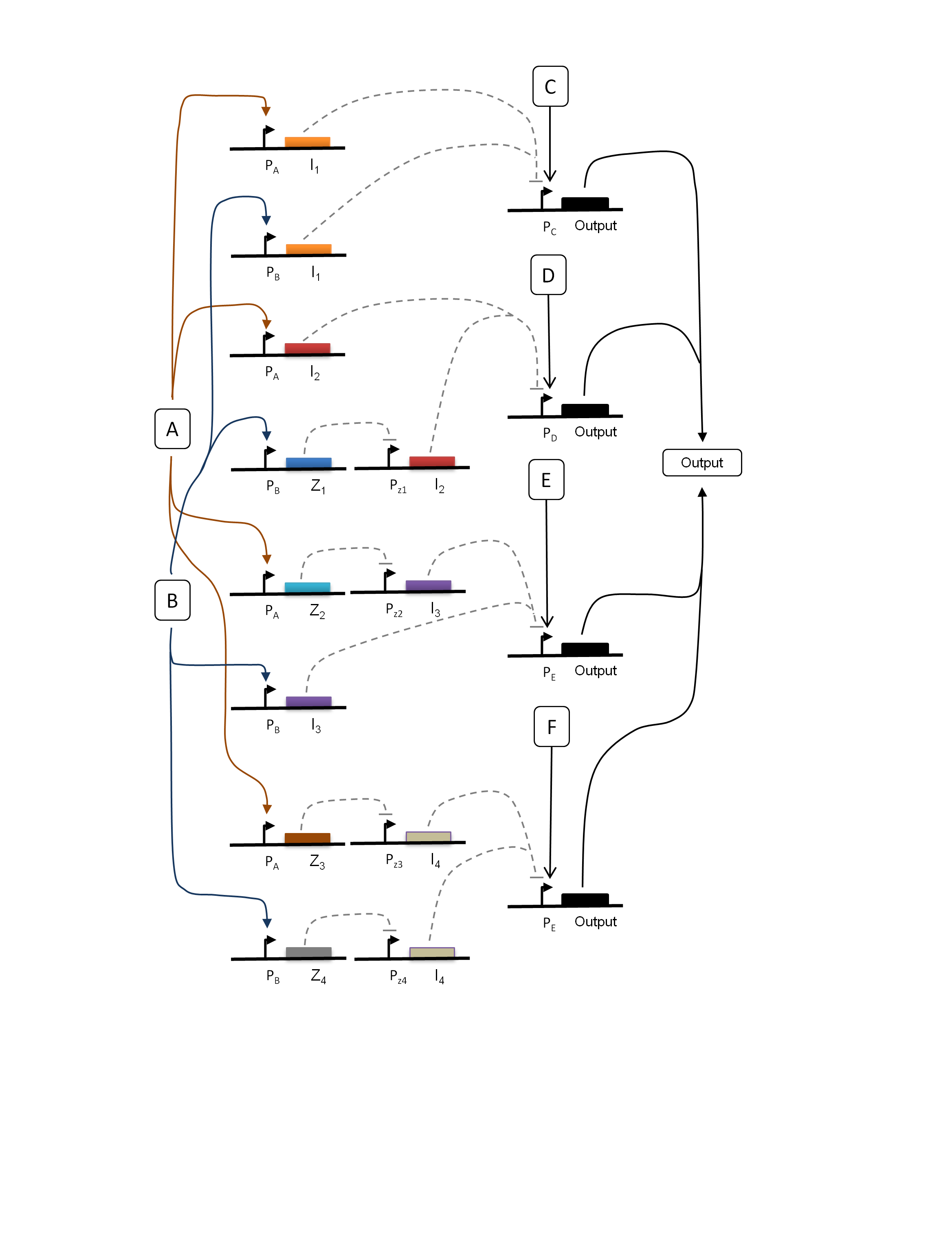

Supplement: S15 Fig — This circuit requires 10 different promoters, 6 regulated by the external inputs A, B, C, D, E, and F, and 4 for internal connections. Additionally, 8 different wires are necessary (dashed lines), implemented by 8 different repressor proteins. (TIF) [file pcbi.1004685.s019.TIF]
